# Supplementary material for: Genomic insights into the conservation status of the world’s last remaining Sumatran rhinoceros populations
Source: Nat Commun. 2021 Apr 26;12:2393. doi: 10.1038/s41467-021-22386-8 (PMC8071806; doi:10.1038/s41467-021-22386-8)
Supplement: Supplementary file 1 — Supplementary Information [file 41467_2021_22386_MOESM1_ESM.pdf]

Supplementary Information

Genomic insights into the conservation status of the world's last remaining Sumatran rhinoceros populations

Johanna von Seth[+*], Nicolas Dussex[+*], David Díez-del-Molino, Tom van der Valk, Verena E. Kutschera, Marcin Kierczak, Cynthia C. Steiner, Shanlin Liu, M. Thomas P. Gilbert, Mikkel-Holger S. Sinding, Stefan Prost, Katerina Guschanski, Senthilvel K.S.S. Nathan, Selina Brace, Yvonne L. Chan, Christopher W. Wheat, Pontus Skoglund, Oliver A. Ryder, Benoit Goossens, Anders Götherström, Love Dalén

[+] These authors contributed equally to this work

[*]Correspondence: johanna.n.vonseth@gmail.com; nicolas.dussex@gmail.com; love.dalen@nrm.se

Supplementary methods

Bioinformatics processing of *de novo* assembly. The *de novo* genome assembly of one male (Kertam; Table S1) Sumatran rhinoceros (*Dicerorhinus sumatrensis harrissoni*; Genbank: https://www.ncbi.nlm.nih.gov/nuccore/JABWHU000000000[1]) was used as reference genome for mapping the re-sequencing data.

In order to identify scaffolds linked to sex chromosomes, we blasted the genome against the horse X chromosome using BLAST+ 2.5.0[2]. The BLAST+ parameters were set as: -evalue = 1e-10; -word_size = 15; -max_target_seqs = 1000. We excluded two putatively X chromosome-linked scaffolds (Sc9M7eS_1319;HRSCAF=1962 and Sc9M7eS_931;HRSCAF=1475) identified through BLAST+ from the assembled genome. For all downstream analyses, we only retained 44 scaffolds >= 1Mb, which represents 99% of the Sumatran rhinoceros genome assembly.

Repeats and transposable elements were *de novo* predicted from the genome assembly using RepeatModeler v.1.0.8[3]. The assembly was subsequently masked in RepeatMasker v.4.0.7[4] using the repeat library from RepeatModeler as input. Finally, we identified CpG sites (all sites where a C nucleotide is followed by a G nucleotide in the reference genome) using a custom script (https://github.com/tvdvalk/find_CpG).

Sample preparation. We obtained 31 historical bone, skin, and tooth samples from Borneo, Sumatra, and the Malay Peninsula collected between 1868 and 1921. We also obtained 16 modern tissue and blood samples from Borneo, Sumatra and the Malay Peninsula.

We gathered approximately 50 mg of tooth or bone powder or 0.5 cm^2 of skin from historical samples and extracted total DNA using a silica-based protocol following Ersmark et al.[5]. To estimate endogenous DNA content (i.e. non-bacterial or contaminant DNA) of historical samples, we constructed double stranded libraries according to Meyer & Kircher[6] and then shotgun-sequenced them at low coverage on one Illumina HiSeq 2500 lane with a 2 × 125 bp setup in the High Output mode at Science for Life Laboratories (SciLifeLab), Stockholm. We then selected five specimens based on high endogenous DNA content (i.e. 36-89%) which was estimated as the proportion of reads mapping to the *de novo* Sumatran rhinoceros assembly (described below).

We extracted DNA from Bornean modern samples consisting of muscle or blood using a Kingfisher robot (Thermo Fisher Scientific) and following the Kingfisher blood &

tissue extraction protocol according to the manufacturer's instructions. DNA, tissue, and cell lines were obtained for all modern samples from Sumatra and Malay Peninsula, as well as for one Bornean sample, from the San Diego Zoo Global Frozen Zoo®. Utilization of samples was compliant with applicable regulatory procedures for CITES and the US Endangered Species Act. DNA was extracted using the DNeasy tissue and cell line kits (Qiagen, CA, USA) according to the manufacturer's instructions. Concentrations were measured using QuBit® 2.0 Fluorometer (Invitrogen, USA) and the quality of the DNA was evaluated by running the samples through agarose gels with electrophoresis.

Library preparation for re-sequencing. For the five historical extracts selected, we built double stranded Illumina libraries according to Meyer & Kircher[6]. We used 20 µl of DNA extract in a 40 µl blunt-end repair reaction with the following final concentration: 1× buffer Tango, 100 µM of each dNTP, 1 mM ATP, 25 U T4 polynucleotide kinase (Thermo Scientific) and 3U USER enzyme (New England Biolabs). Treatment with USER enzyme was performed to excise uracil residues resulting from post-mortem damage[7,8]. Samples were incubated for 3 h at 37°C, followed by the addition of 1 µl T4 DNA polymerase (Thermo Scientific) and incubation at 25°C for 15 min and 12°C for 5 min. The samples were then cleaned using MinElute spin columns following the manufacturer's protocol and eluted in 20 µl EB Buffer. Next, we performed an adapter ligation step where DNA fragments within each library were ligated to a combination of incomplete, partially double-stranded P5- and P7-adapters (10 µM each). This reaction was performed in a 40 µl reaction volume using 20 µl of blunted DNA from the clean-up step and 1 µl P5-P7 adapter mix per sample with a final concentration of 1× T4 DNA ligase buffer, 5% PEG-4000, 5U T4 DNA ligase (Thermo Scientific). Samples were incubated for 30 minutes at room temperature and cleaned using MinElute spin columns as described above. Next, we performed an adapter fill-in reaction in 40 µl final volume using 20 µl adapter ligated DNA with a final concentration of 1× Thermopol Reaction Buffer, 250 µM of each dNTP, 8U Bst Polymerase, Long Fragments. The libraries were incubated at 37°C for 20 minutes and heat-inactivated at 80°C for 20 minutes. These libraries were then used as stock for indexing PCR amplification. In order to increase library complexity, six to ten indexing PCR amplifications were performed for each library using different P7 indexing primers[6]. Amplifications were performed in 25 µl volumes with 3 µl of adapter-ligated library as template, with the following final concentrations: 1x AccuPrime reaction mix, 0.3 µM IS4 amplification primer, 0.3 µM P7 indexing primer, 7 U

AccuPrime Pfx (Thermo Scientific) and the following cycling protocol: 95°C for 2 min, 10-14 cycles at 95°C for 30 s, 55°C for 30 s and 72°C for 1 min and a final extension at 72°C for 5 minutes.

Purification and size selection of libraries was then performed using Agencourt AMPure XP beads (Beckman Coulter, Brea, CA, USA), first using 0.5X bead:DNA ratio and second 1.8X to remove long and short (i.e. adapter dimers) fragments, respectively. Library concentration was measured with a high-sensitivity DNA chip on a Bioanalyzer 2100 (Agilent, Santa Clara, CA, USA). Finally, multiplexed libraries (i.e. six to ten indexed libraries) were pooled into a single pool in equimolar concentrations and sequenced on an Illumina HiSeqX with a 2 × 150 bp setup in the High Output mode at the National Genomics Infrastructure Sweden (NGI) at Science for Life Laboratories (SciLifeLab), Stockholm.

The 16 modern DNA extracts were submitted to NGI at SciLifeLab (Stockholm). Illumina TruSeq PCR-free libraries were prepared for 12 high DNA concentration samples while Lucigen NxSeq AmpFREE Low and Rubicon ThruPLEX DNA-seq construction were prepared for the four modern extracts with lower DNA concentration. These libraries were subsequently re-sequenced on an Illumina HiSeqX with a 2 × 150 bp setup in the High Output mode.

Bioinformatics processing of re-sequencing data. All data processing and analyses of re-sequencing data, as well as an initial mate-pair assisted assembly, were performed on resources provided by the Swedish National Infrastructure for Computing (SNIC) at UPPMAX, Uppsala University. Raw historical sequence data were demultiplexed using bcl2Fastq v2.17.1 with default settings (Illumina Inc.). SeqPrep v.1.1[9] was then used to trim adapters and merge paired-end reads, using default settings but with a minor modification in the source code, allowing us to choose the best quality scores of bases in the merged region instead of aggregating the scores, following Palkopoulou et al.[10]. As recommended for historical and ancient DNA short reads[11], we merged sequencing reads and mapped them against the *de novo* reference genome for Sumatran rhinoceros (*D. sumatrensis harrissoni*; Genbank: https://www.ncbi.nlm.nih.gov/nuccore/JABWHU000000000[1]) using the BWA v.0.7.13 aln algorithm[12] and used slightly modified settings that deactivated seeding (-l 16,500), allowed more substitutions (-n 0.01) and allowed up to two gaps (-o 2). The BWA 'samse' command was then used to generate alignments in SAM format. The resulting reads were then processed in SAMtools v.1.3[13] and converted to BAM format, as well as

coordinate-sorted and indexed. We removed duplicates from the alignments using a modified Python script (https://github.com/pontussk/samremovedup) from Palkopoulou et al.[10] in order to avoid inflation of length distribution for loci with deep coverage.

For modern samples, forward and reverse reads were trimmed to remove Illumina adapter sequences using Trimmomatic v.0.32 with default settings[14] and then mapped to the reference genome using BWA mem v.0.7.13[12]. SAMtools v.1.3[13] was used for coordinate sorting, indexing, and removing duplicates from the alignments.

Historical and modern alignments were then processed in parallel. We used Picard v.1.141 (http://broadinstitute.github.io/picard) to assign read group information including library, lane and sample identity to each bam file. Reads were then re-aligned around indels using GATK IndelRealigner v.3.4.0[15]. Only read alignments with mapping quality ≥30 were kept for subsequent analysis. Finally, we estimated the average depth of genome coverage using SAMtools[16]. Three specimens (SR01, OR2142 and Gelugob) had very low average depth of coverage of 3, 5, and 2X and were excluded from all analyses except the structure analyses (i.e. PCA and ADMIXTURE). After excluding these three low-coverage samples, the average depth of genome coverage ranged from 17 and 29X in modern and from 9X to 13X for historical genomes.

We called variants in historical and modern genomes using bcftools mpileup v.1.3[13] and bcftools call v.1.3. Resulting vcf files were filtered, keeping sites with a minimum depth of coverage of 1/3X of the average coverage, base quality ≥30 and excluding SNPs within ± 5bp of indels. We also excluded CpG sites and repeat regions identified in the reference genome (see 'Bioinformatics processing of *de novo* assembly' section) using BEDtools v.2.27.1[17]. For all downstream analyses, we excluded two X chromosome-linked scaffolds (Sc9M7eS_1319;HRSCAF=1962 and Sc9M7eS_931;HRSCAF=1475; Section 2) from the vcf files. After filtering, 98,73,772 SNPs were identified across all individuals. We then used PLINK v.1.9[18] to further filter this dataset and remove genotypes missing from more than 10% of the samples (--geno 0.1). We first performed this filtering for all 21 rhinoceros, which yielded 3,568,319 high quality SNP calls and used this dataset for population structure analyses (i.e. PCA, Admixture). Second, we performed the same filtering step only for the 18 rhinoceros with coverage ≥9X and removed all missing genotypes (--geno 0). We obtained 4,656,534 SNPs that were used for all other analyses (genome-wide diversity, inbreeding, mutational load, variants in coding regions, tests of positive selection).

Data analysis

Population structure. We first built an unrooted phylogeny. We inferred genotype posterior probabilities for each individual using ANGSD v.0.921[19] from bam files, then used ngsDist[20] to estimate pairwise genetic distances directly from the genotype posterior probabilities, with 100 bootstrap replicates. Next, we estimated a phylogeny from the distance matrix using FASTME v.2.0[20,21].

Second, we used the SmartPCA from the EIGENSOFT v5.0[22,23] package to perform a principal component analysis (PCA) using the filtered SNP dataset for 21 rhinoceros samples (autosomes only). The variant files were converted to the eigenstrat format using the python script 'vcf2eigenstrat.py' from gdc (https://github.com/mathii/gdc.git).

Third, we used the ADMIXTURE v.1.3.0[24] software to identify genetic clusters K ranging from 1 to 6. This program estimates ancestry in a model-based manner where individuals are considered unrelated and uses a cross-validation procedure to determine the best number of possible genetic groups present in the dataset.

For both these analyses, one historical specimen (SR22) labelled as originating from the Sumatran Coast clustered with the Malay Peninsula and the captive born specimen from Cincinnati Zoo (KB20219, offspring of KB7902 and KB9342) clustered with other Sumatran samples. These two specimens were thus grouped with other samples from their respective genetic cluster.

Demographic reconstruction and population divergence. We inferred the temporal fluctuations in the effective population sizes (N_e) of the three major lineages of Sumatran rhinoceros using the Pairwise Sequentially Markovian Coalescent (PSMC v.0.6.5)[25]. This approach infers the time to the most recent common ancestor (TMRCA) between independent segments of the genome. Regions of low heterozygosity indicate recent coalescent events while regions of high heterozygosity indicate more ancient coalescent events. Moreover, because the rate of coalescence is inversely proportional to N_e, it can then be used to estimate temporal changes in N_e. We generated consensus sequences for all autosomes of historical and a subset of the modern genomes using the Samtools mpileup v.1.3[13,25] command and the 'vcf2fq' command from vcfutils.pl. We used filters for base quality, mapping quality and root-mean-squared mapping quality below 30, and depth below 1/3 and higher than 2-times the average depth of genome coverage estimated for each specimen. In order to infer the

distribution of the time to the most recent common ancestor (TMRCA) between the two copies of each chromosome from each individual across all autosomes, we set N (the number of iterations) = 30, t (Tmax) = 15 and p (atomic time interval) = 64 (4 + 25 * 2 + 4 + 6, for each of which parameters are estimated with 28 free interval parameters), and did bootstrap tests with 100 replicates. We used the intermediate substitution rate of 2.34×10^{-8} substitutions/site/generation from the ones compared in Mays et al.[26] and a generation time of 12 years[27].

We then estimated the split time (T) between each pair of Sumatran rhinoceros populations by assuming no coalescent events since divergence between the populations and using the PSMC approach applied to a pseudo-diploid X chromosome genome. Because the PSMC infers the TMRCA between two alleles carried by one individual across its genome, this method can be used in a similar way to infer the TMRCA between two haploid genomes from two populations[25,28]. Because the estimated N_e is inversely proportional to the coalescence rate between two chromosomes, an extremely large N_e reaching infinity should indicate a period of isolation between two ancestral populations. We first extracted the two X-chromosome-linked scaffolds from bam files of one male per population (Kertam from Borneo, KB7902 from Sumatra, and SR08 from Malay Peninsula). We then generated X-chromosome haploid consensus sequences for these three males and merged each pair combination into a pseudo-diploid X chromosome sequence using the seqtk mergefa command. Next, we applied the PSMC method on the pseudo-diploid X chromosome to estimate changes in N_e over time. Finally, we rescaled the pseudo-diploid X chromosome curve to 0.25 consistent with the effective population size of chromosome X relative to that of autosomes (sex-chromosome/autosome ratio: 0.75). For all three pairwise population comparisons, we ran the analysis using the same quality filters, parameters (i.e. 64 discrete time intervals) and the same substitution rate as above for the PSMC on autosomes. In order to avoid underestimation of the split time, we also ran the same analysis using fewer discrete intervals (i.e. 49 = 6 + 4 + 3 + 13 * 2 + 4 + 6 or 37 = 2 + 2 + 1 + 15 * 2 + 2) as recommended by Prado-Martinez et al.[28].

Genome-wide heterozygosity and Runs of Homozygosity (ROH). For these analyses, we included the 18 individuals with an average depth of genome coverage ≥9X. We estimated the individual autosomal heterozygosity using mlRho v.2.7[29]. mlRo allows us to estimate the population mutation parameter (θ), which approximates the per site heterozygosity under the

infinite sites model. We filtered out positions with base quality <30 and root-mean-squared mapping quality <30, as well as reads with mapping quality <30. Because high or low coverage in some regions resulting from structural variation can create erroneous mapping to the reference genome and false heterozygous sites, for each specimen, we filtered out sites with depth lower than 1/3X and higher than 10X the average coverage across all our specimens. The maximum likelihood approach implemented in mlRho has been shown to provide unbiased estimates of average within-individual heterozygosity at high coverage[29,30]. We statistically compared θ between groups using two-sided pairwise t-tests in R v.3.3.3[31].

We then identified runs of homozygosity (ROH) and estimated individual inbreeding coefficients (F_{ROH}) using two different sliding-window approaches.

We first converted the filtered multi-individual vcf file (i.e. 4,656,534 SNPs as described above) into a .ped file and identified ROH in PLINK v.1.9[18]. To assess the robustness of our results to the applied parameters and to potential sequencing errors, we used three different sets of parameters where we varied the window size (*homozyg-window-snp*) and the number of heterozygous site per window (*homozyg-window-het*) such as: 1) *homozyg-window-snp* 100 and *homozyg-window-het* 1; 2) *homozyg-window-snp* 250 and *homozyg-window-het* 3 (reported in main text in Figs. 3 and 4); 3) *homozyg-window-snp* 500 and *homozyg-window-het* 5. All other parameters described hereafter were the same for each of the three parameter sets. If at least 5% of all windows that included a given SNP were defined as homozygous, the SNP was defined as being in a homozygous segment of a chromosome (*homozyg-window-threshold 0.05*). This threshold was chosen to ensure that the edges of a ROH are properly delimited. A homozygous segment was then defined as a ROH if all of the following conditions were met: the segment included ≥25 SNPs (*homozyg-snp 25*); the segment covered ≥100 kb (*homozyg-kb* 100); the minimum SNP density was one SNP per 50 kb (*homozyg-density 50*); the maximum distance between two neighbouring SNPs was ≤1,000 kb (*homozyg-gap 1,000*); the number of heterozygous sites within ROH was set to 750 (*homozyg-het 750*) in order to prevent sequencing errors to cut ROH.

Second, we used ROHan to obtain independent support for our ROH analysis[32]. In contrast to PLINK, ROHan runs a hidden-markov model, jointly estimating genome wide heterozygosity and ROH identification and as such does not require user defined input settings.

Based on these results, we then estimated the individual inbreeding coefficient F_{ROH} as the overall proportion of the genome contained in ROH for 1) ROH ≥100 kb ($F_{ROH\geq}$100 kb) and 2) ROH ≥2 Mb (F_{ROH} ≥2 Mb). ROH ≥100 kb are indicative of background relatedness while ROH ≥2 Mb indicate recent mating between related individuals[33].

Finally, we statistically compared F_{ROH} between groups using two-sided pairwise t-tests in R v.3.3.3[31].

Mutational load based on evolutionary constrained regions. Here, we used an estimate of genome conservation across evolutionary time, measured by GERP-scores, as a proxy for the deleteriousness of a given genomic variant. We measured mutational load in each individual as the number of homozygous and heterozygous derived alleles at sites that are under strict evolutionary constraints using genomic evolutionary rate profiling scores (GERP) with the GERP++ software[34]. GERP identifies constrained elements in multiple alignments by quantifying the amount of substitution deficits (e.g. substitutions that would have occurred if the element was neutral, but did not occur because the element has been under functional constraint) by accounting for phylogenetic divergence. High GERP scores (>4) represent highly conserved regions whereas low scores (<1) are putatively neutral.

To identify the highly conserved regions in the Sumatran rhinoceros genome we first obtained 231 published mammalian reference genomes from NCBI (https://www.ncbi.nlm.nih.gov/assembly/organism/40674/all/) and used TimeTree (http://www.timetree.org/) to obtain divergence times based on automated literature searches[35]). Each of these genomes was then converted into fastq-format (50 bp reads) and realigned against the Sumatran rhinoceros reference using bwa-mem v.0.7.13[12], with slightly lowered mismatch and gaps penalty scores (-B 3, -O 4,4). Additionally, we filtered out all reads aligning to more than one genomic location using Samtools v.1.3[13]. Next, we converted each alignment file to fasta-format using htsbox v.1.0 -R -q 30 -Q 30 -l 35 -s 1, https://github.com/lh3/htsbox). GERP++ was then used to calculate conservation scores for each site in the genome for which at least 3 mammal species could be accurately aligned to the Sumatran rhinoceros reference genome. We used the genome alignment and annotation of the white rhinoceros (*Ceratotherium simum simum*; Genbank: https://www.ncbi.nlm.nih.gov/assembly/GCF_000283155.1/) reference to infer ancestral alleles and GERP-scores. As expected, we found higher GERP-scores within exons than outside exons, supporting that this method accurately estimates genome conservation.

However, some overlap could be seen (i.e. not all exonic regions are highly conserved whereas some putatively non-coding regions are).

Next, for each individual we obtained the total number of derived alleles stratified by GERP-score within highly conserved regions of the genome (excluding sites with missing genotypes) as a proxy of mutational load. We also estimated the individual relative mutational load measured as the sum of all derived alleles multiplied by their GERP-score, only including derived alleles above GERP-score of 4, divided by the total number of derived alleles per individual.

We also calculated the percentage of derived alleles unique to each population or shared between populations at high GERP-score (>4), e.g. those putatively deleterious. We randomly subsampled six alleles at each genomic site with a GERP-score above 4, from each of the modern populations (thus three samples per population to exclude sample biases) and counted how often a derived allele was unique to a specific population or shared with one or both of the other populations.

Finally, we statistically compared individual relative mutational load between groups using two-sided pairwise t-tests in R v.3.3.3[31].

Mutational load in coding regions and missense variants. We annotated synonymous and non-synonymous nucleotide substitutions within coding regions as well as substitutions in proximity of coding regions for modern and historical Sumatran rhinoceros using SNPeff v.4.3[36]. In order to avoid reference and annotation bias, we mapped 18 resequenced genome with a coverage ≥9X (i.e. excluding three low-coverage genomes: SR01, OR2142, and Gelugob) to the white rhinoceros genome (*C. simum simum*; Genbank: https://www.ncbi.nlm.nih.gov/assembly/GCF_000283155.1/) using the same mapping and variant calling parameters as described above (see 'Bioinformatics processing of re-sequencing data' section) and used its annotation when annotating variants. After filtering for missing data we obtained 13,157,914 SNPs.

First, we generated a database for white rhinoceros using the protein sequences extracted from its annotation. We used the -V option of gffread from the cufflinks v.2.2.1[37,38] package to remove in-stop codons from the annotation and obtained a total of 33,026 genes. Second, we identified variants in three different categories as described in the SNPeff manual: a) *Synonymous*: mostly harmless or unlikely to change protein behaviour; b) *Missense*: non-disruptive variants that might change protein effectiveness; c) Loss-of-Function (LoF): variants assumed to have high (disruptive) impact on the protein,

probably causing protein truncation, loss of function (LoF) or triggering nonsense mediated decay (e.g. stop codons, splice donor variant and splice acceptor)[36]. We also differentiated variants in these four categories separated by homozygous and heterozygous state.

We performed two types of comparisons: 1) between the modern and historical specimens for Borneo (n = 5) and the Malay Peninsula (n = 6), and 2) among modern samples from the Bornean (n = 4), Malay Peninsula (n = 3) and Sumatran (n = 8) populations. We then compared the number of variants among populations using two-sided pairwise t-tests in R v.3.3.3[39]. For the comparison among modern samples, we reported the number of LoF variants shared among and unique to each population and estimated the difference in frequency of LoF variants among populations in PLINK v.1.9 [18].

Finally, we used the per-individual identified LoF variants to predict the risk of introducing new LoF variants in a receiving population in the case of translocation of individuals. To do this we counted the number of LoF variants in each individual, then estimated how many of them were absent (allele frequency = 0) in the other two populations.

Detecting positive selection. First, for each population we estimated the frequency of variants characterised as missense to identify genes potentially involved in local adaptation in modern populations and statistically compared the number of variants among populations as described in the previous section. We also reported the number of missense variants common or unique to each population.

Second, we used the Population Branch Statistic (PBS) to investigate adaptation to local environments in the three Sumatran rhinoceros populations. PBS is a statistic based on log-transformed F_{ST} differences (method-of-moments[40]) between a target population and a sister population, but it also incorporates the F_{ST} distance between each of those populations and a third population[41]. This allows the estimation of the amount of divergence specific to the branch leading to the target population since its divergence from both the sister and the other population. Therefore, an extremely divergent branch in the target population can be considered as a signal of selection for that specific locus. This method has demonstrated larger power to detect loci under selection in comparison to other statistics based on measures such as allele frequency alone[41,42].

We used ANGSD v.0.921[19] to estimate the PBS for each one of the 33,026 high-quality gene models in the Bornean, Sumatran and Malay Peninsula populations. For each gene, we first estimated the allele frequency likelihood of all sites in each population excluding reads with mapping quality <30, bases with quality <30, read depth <3 per sample,

and keeping only sites that fulfil these parameters in *n* - 1 minimum of samples, where *n* is the number of samples per population (including historical samples). We then estimated the 2dSFS for each pair of populations and used *realSFS* to estimate the PBS value per gene for each population. Given our limited sample size, PBS values can get large due to extreme allele frequency differences. For example, if a gene's F_{ST} between the target and the sister population is 1, with both populations fixed for a different allele, the PBS value can be infinite. Thus, we replaced infinite PBS values with the maximum value of the distribution of PBS values for that population. Finally, we reported all genes with a larger PBS value than 3, ca. the 99.8th quartile of the distribution of the PBS values of all genes for each population.

Gene Ontology analysis. We assessed the biological functions associated with LoF and missense variants as well as for genes identified with the PBS approach and tested for statistical overrepresentation for each of these categories. We ran this analysis in Panther[43] and used horse (*Equus caballus*) as a reference set. We ran a test of statistical overrepresentation using a Fisher exact test and a False Discovery rate of 0.05.

Supplementary figures

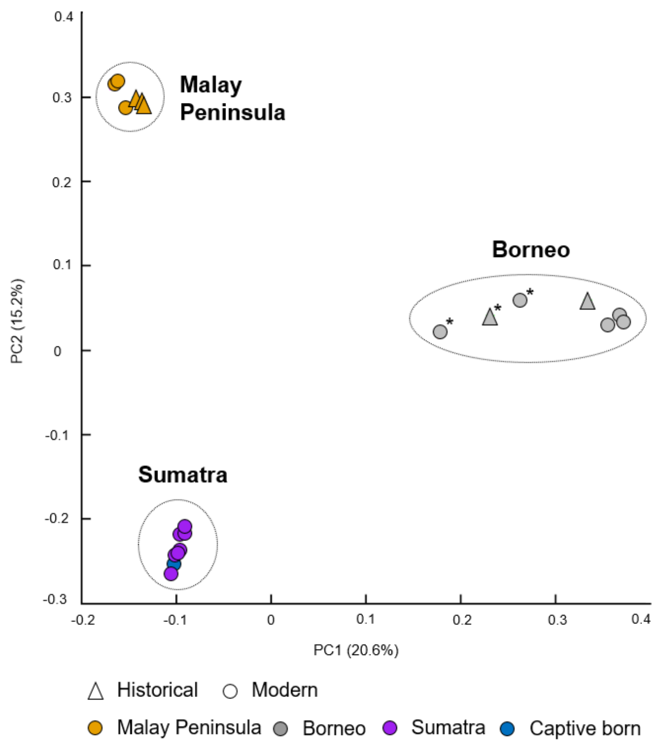

Supplementary Figure 1. Principal Component Analysis for the 21 Sumatran rhinoceros samples. Asterisks represent low coverage (<9X) genomes.

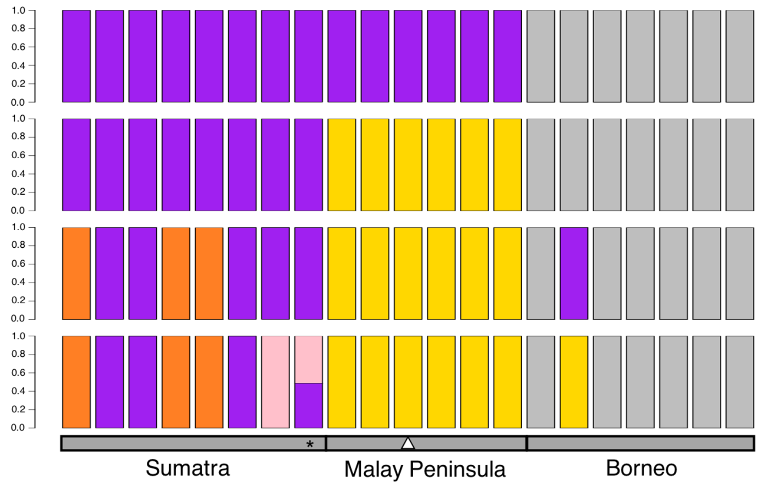

Supplementary Figure 2. Individual clustering assignment of the 21 Sumatran rhinoceros using 3,568,319 SNPs. The lowest cross-validation error was obtained for K=3. For K=4 and K=5, two individuals color coded in orange originate from northeastern Sumatra (the specific location of the third individual is unknown) and three individuals color coded in purple or rose originate from southwestern Sumatra (the specific location of the fourth individual is unknown). The asterisk and triangle depict the captive born rhinoceros (KB20219), whose parents are both from southwestern Sumatra, and the mislabelled museum specimen (SR22), respectively.

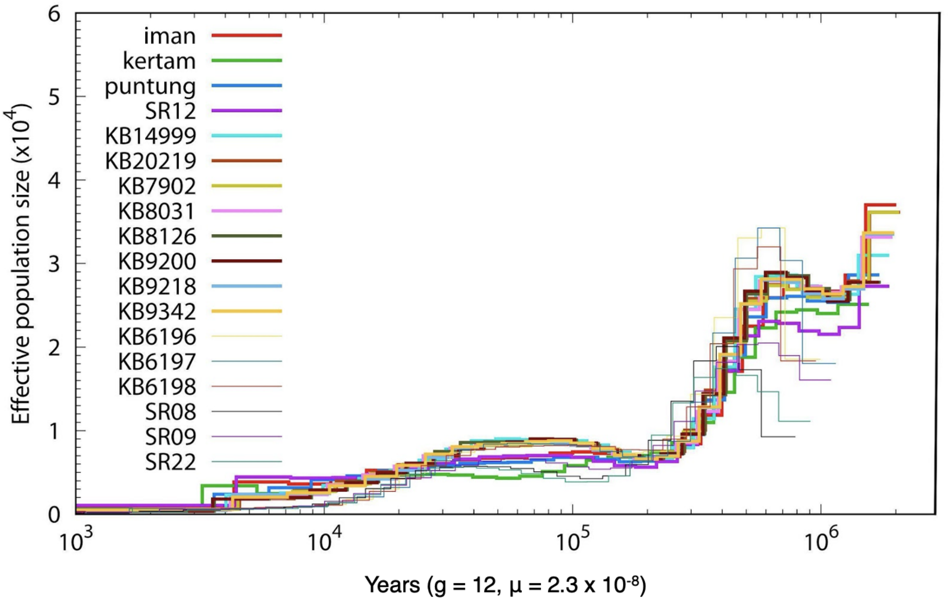

Supplementary Figure 3. Population history for 18 modern and historical Sumatran rhinoceros individuals. Each coloured curve depicts temporal fluctuations in N_e for one individual (see Supplementary Table S1 for the origin of the individual samples). The x-axis corresponds to time before present in years on a log scale, assuming a substitution rate of 2.34×10^{-8} substitutions/site/generation[26,44] and a generation time of 12 years[27]. The y-axis corresponds to the effective population size N_e.

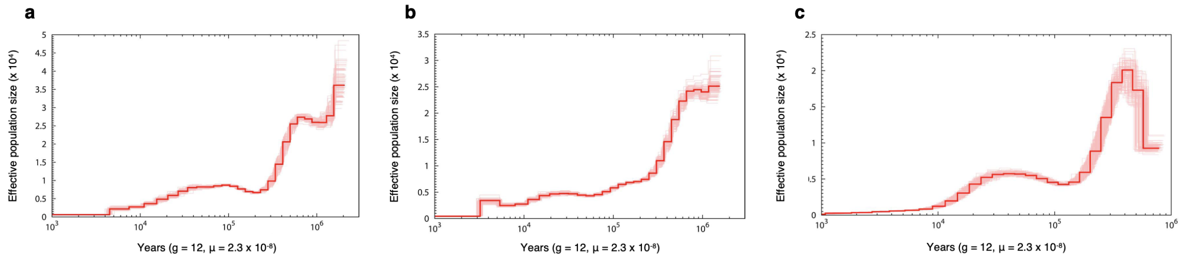

Supplementary Figure 4. Population history for three Sumatran rhinoceros individuals including bootstrap tests with 100 replicates. The thick red line corresponds to the inferred change in N_e and thin bright red lines corresponds to bootstrap tests (100 replicates). (a) Kertam, a modern sample from the Bornean population, (b) SR08, a historical sample from the Malay Peninsula population, and (c) KB7902, a modern sample from the Sumatran population. The x-axis corresponds to time before present in years on a log scale, assuming a substitution rate of 2.34×10^{-8} substitutions/site/generation[26,44] and a generation time of 12 years[27]. The y-axis corresponds to the effective population size N_e.

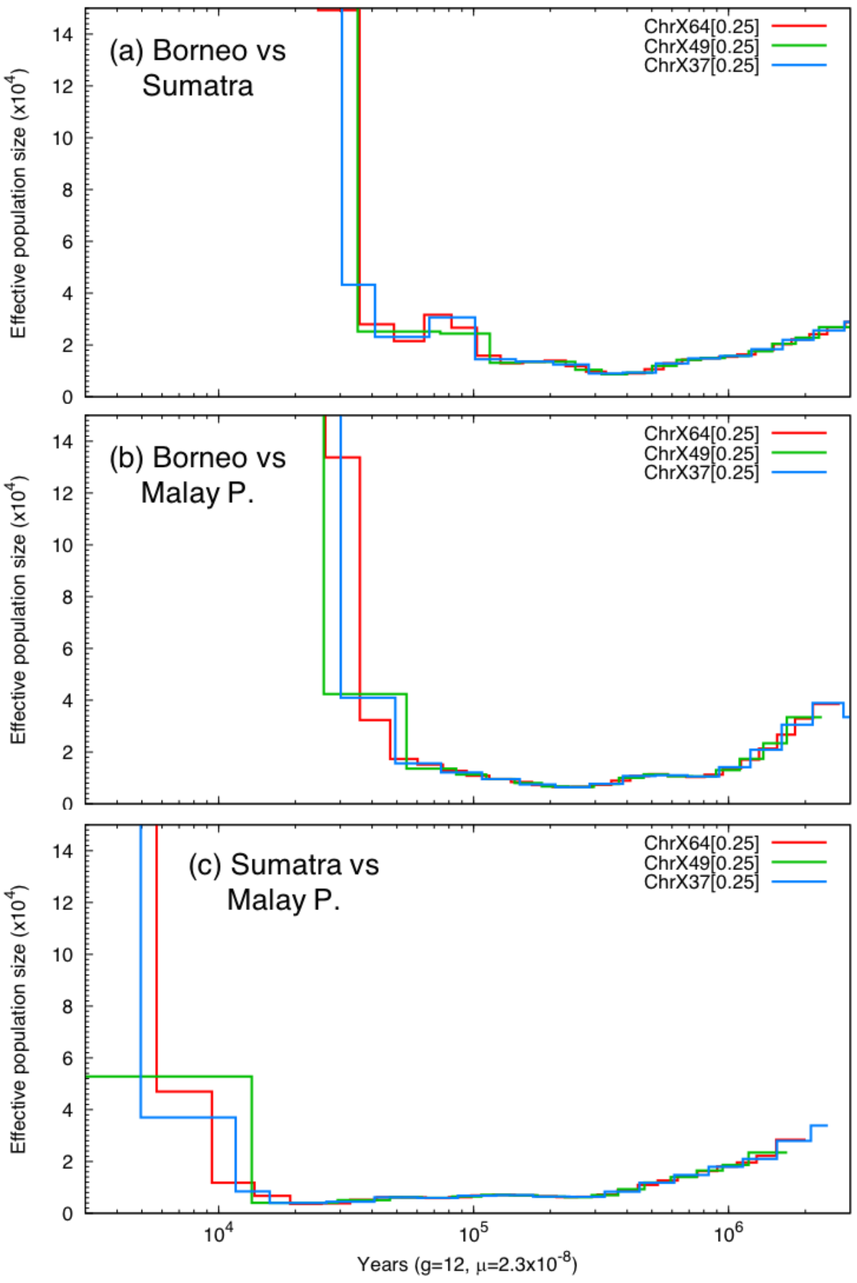

Supplementary Figure 5. Divergence time estimates between Sumatran rhinoceros populations using the PSMC approach. (a) Borneo vs Sumatra. **(b)** Borneo vs Malay Peninsula. **(c)** Sumatra vs Malay Peninsula. The x-axis corresponds to time before present in years on a log scale, assuming a mutation rate of 2.34×10^{-8} substitutions/site/generation[26,44]and a generation time of 12 years[27]. The red, green and blue curves represent the pseudo-diploid chromosome X genome rescaled by factor of 0.25 (sex-chromosome/autosome ratio: 0.75) and using 64, 49 and 37 discrete intervals, respectively. The time where population size goes to infinity (i.e. vertical line) corresponds to time of divergence between the two populations considered.

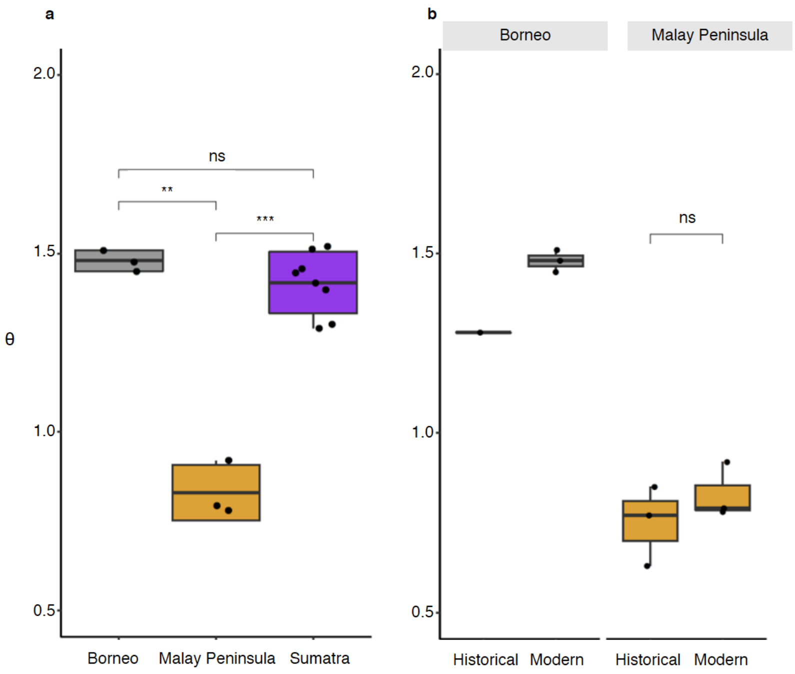

Supplementary Figure 6. Genome-wide autosomal heterozygosity per 1000 bp approximated from the population mutation rate θ. (a) Comparison between modern-day populations (two-sided pairwise t-test, n = 14, $p_{Borneo\text{-}MalayP}$ = 1.1e-06, $p_{Borneo\text{-}Sumatra}$ = 0.2, $p_{MalayP\text{-}Sumatra}$ = 7.1e-07). **(b)** Temporal comparison between historical and modern genomes in the Bornean and Malay Peninsula populations (two-sided t-test, n = 6, p = 0.37). Middle thick line within boxplots and bounds of boxes represent mean and standard deviation, respectively. Vertical lines represent minima and maxima. *** = p <0.001, ** = p <0.01, ns = non-significant, p-values were not adjusted for multiple comparisons.

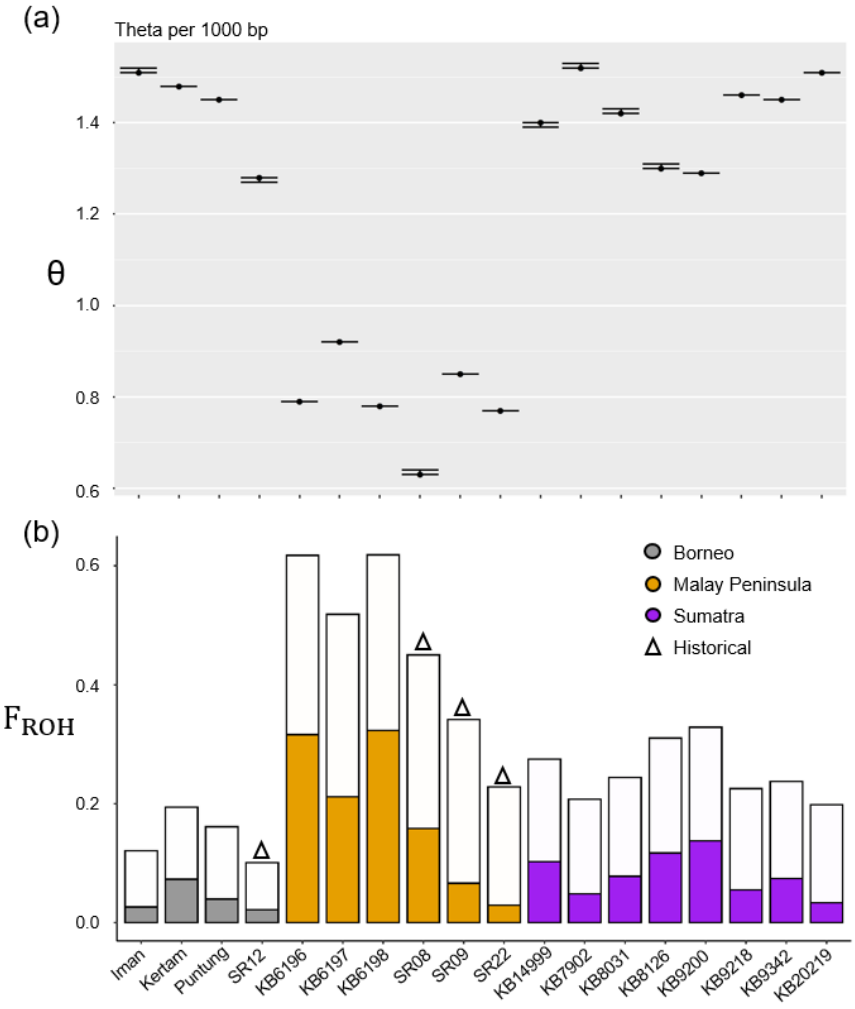

Supplementary Figure 7. **Individual heterozygosity and inbreeding coefficients. (a)** Individual autosomal heterozygosity per 1,000 bp estimated as the population mutation rate θ (n = 18). Bars extending from the black points represent standard deviation. **(b)** Individual inbreeding coefficients estimated as the average proportion of the genome in F_{ROH}. Empty bars show the portion of the genome contained in ROH ≥100 kb and full bars the portion of the genome contained in ROH ≥2 Mb. ROH were estimated using the following parameters (see text): homozyg-window-snp 250 and homozyg-window-het 3. Triangles depict historical specimens.

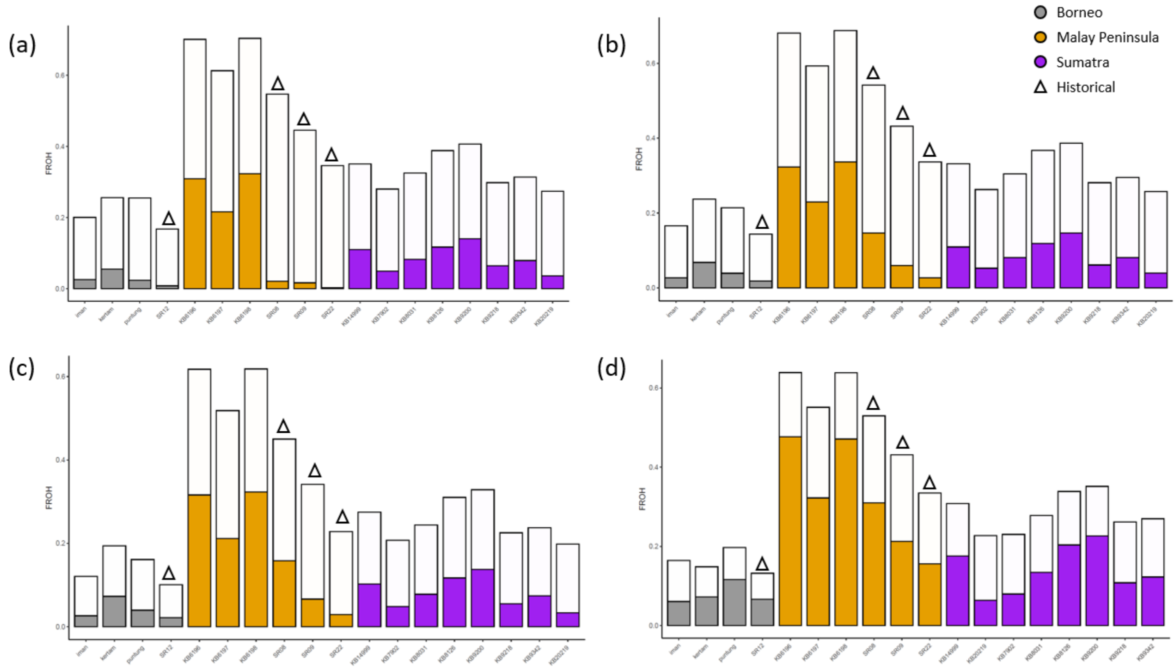

Supplementary Figure 8. Comparison of estimated individual F_{ROH} between three different sets of parameters in PLINK and ROHan. For the PLINK analyses (a-c) we varied the window size (homozyg-window-snp) and the number of heterozygous site per window (homozyg-window-het) such as: **(a)** homozyg-window-snp 100 and homozyg-window-het 1; **(b)** homozyg-window-snp 250 and homozyg-window-het 3; **(c)** homozyg-window-snp 500 and homozyg-window-het 5; **(d)** ROHan.

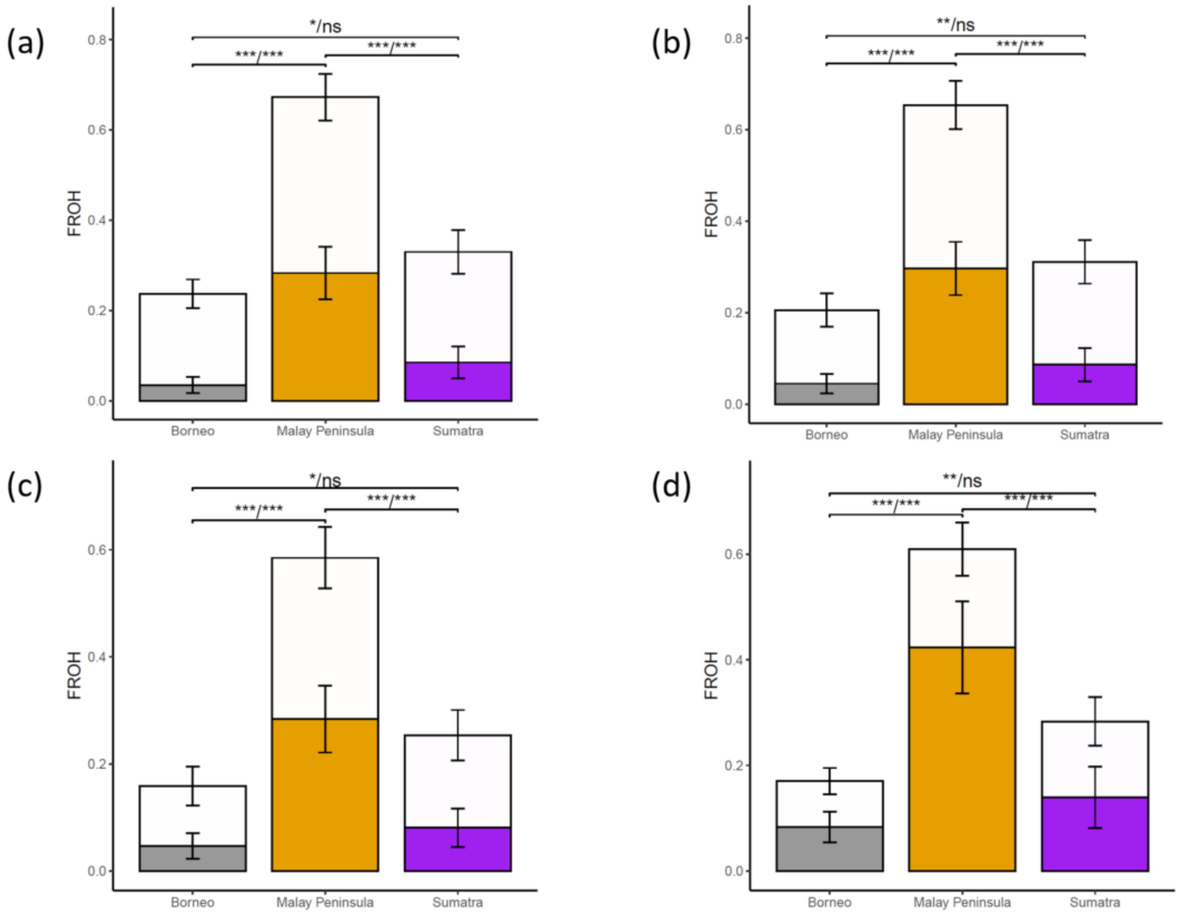

Supplementary Figure 9. Comparison of estimated F_{ROH} in modern populations between three different sets of parameters in PLINK and ROHan. For the PLINK analyses (a-c) we varied the window size (homozyg-window-snp) and the number of heterozygous site per window (homozyg-window-het) such as: **(a)** homozyg-window-snp 100 and homozyg-window-het 1 (two-sided pairwise t-test, F_{ROH} ≥100 kb: $p_{Borneo\text{-}MalayP}$ = 5.8e-07, $p_{Borneo\text{-}Sumatra}$ = 0.014, $p_{MalayP\text{-}Sumatra}$ = 6.5e-07; F_{ROH} ≥ 2 Mb: $p_{Borneo\text{-}MalayP}$ = 2.2e-05, $p_{Borneo\text{-}Sumatra}$ = 0.081, $p_{MalayP\text{-}Sumatra}$ = 2.2e-05); **(b)** homozyg-window-snp 250 and homozyg-window-het 3 (also reported in main text) (two-sided pairwise t-test, F_{ROH} ≥100 kb: $p_{Borneo\text{-}MalayP}$ = 4.2e-07, $p_{Borneo\text{-}Sumatra}$ = 0.0066, $p_{MalayP\text{-}Sumatra}$ = 6.4e-07; F_{ROH} ≥ 2 Mb: $p_{Borneo\text{-}MalayP}$ = 2.2e-05, $p_{Borneo\text{-}Sumatra}$ = 0.15, $p_{MalayP\text{-}Sumatra}$ = 2.2e-05); **(c)** homozyg-window-snp 500 and homozyg-window-het 5 (two-sided pairwise t-test, F_{ROH} ≥100 kb: $p_{Borneo\text{-}MalayP}$ =8.8e-07, $p_{Borneo\text{-}Sumatra}$ = 0.014, $p_{MalayP\text{-}Sumatra}$ = 1.1e-06; F_{ROH} ≥ 2 Mb: $p_{Borneo\text{-}MalayP}$ = 4e-05, $p_{Borneo\text{-}Sumatra}$ = 0.23, $p_{MalayP\text{-}Sumatra}$ = 4e-05); **(d)** ROHan (two-sided pairwise t-test, F_{ROH} ≥100 kb: $p_{Borneo\text{-}MalayP}$ = 9.6e-08, $p_{Borneo\text{-}Sumatra}$ = 0.003, $p_{MalayP\text{-}Sumatra}$ = 3.0e-07; F_{ROH} ≥ 2 Mb: $p_{Borneo\text{-}MalayP}$ = 2.7e-05, $p_{Borneo\text{-}Sumatra}$ = 0.2, $p_{MalayP\text{-}Sumatra}$ = 2.6e-05). *n* = 14, *** = $p < 0.001$, ** = $p < 0.01$, ns = non-significant, p-values were not adjusted for multiple comparisons, error bars represent the standard deviation.

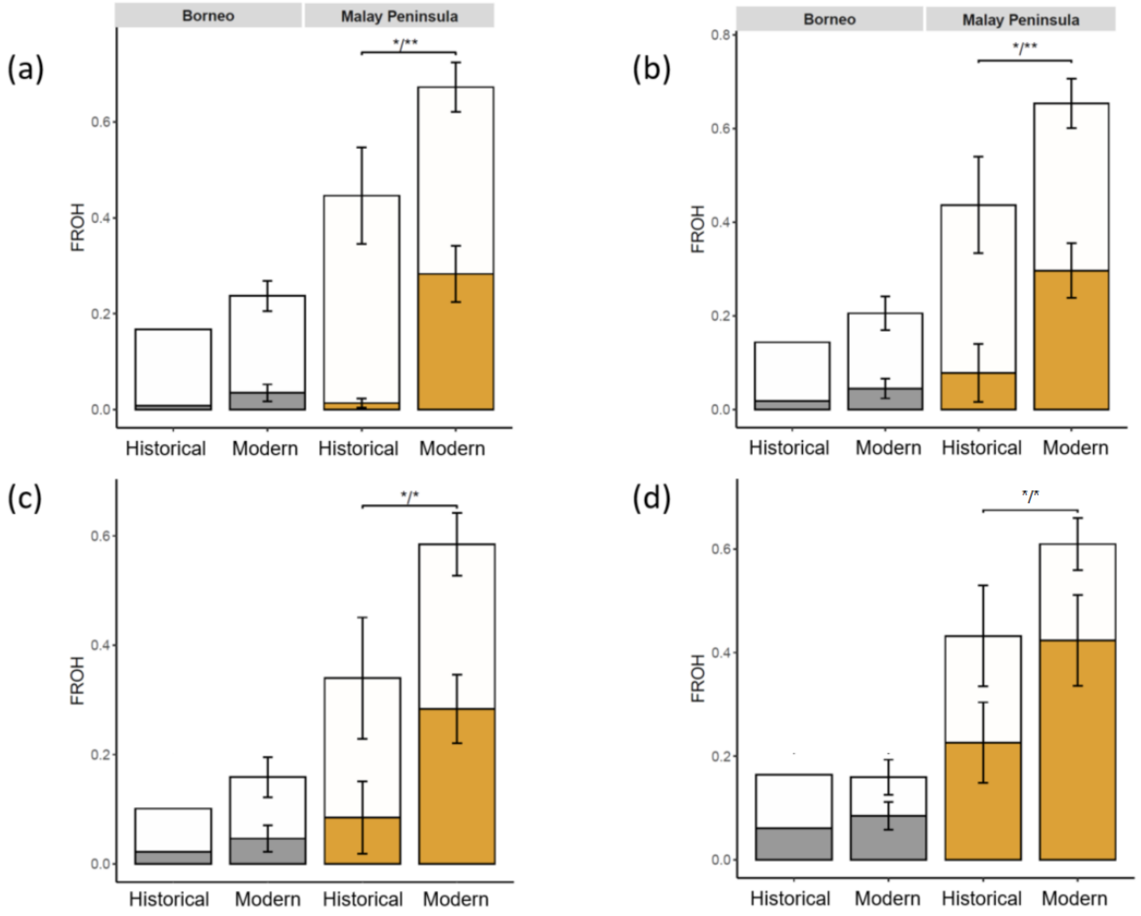

Supplementary Figure 10. Comparison of estimated temporal changes in F_{ROH} between three different sets of parameters in PLINK and ROHan. For the PLINK analyses (a-c) we varied the window size (homozyg-window-snp) and the number of heterozygous site per window (homozyg-window-het) such as: **(a)** homozyg-window-snp 100 and homozyg-window-het 1 (two-sided t-test, F_{ROH} ≥100 kb: p = 0.0315, F_{ROH} ≥ 2 Mb: p = 0.00139); **(b)** homozyg-window-snp 250 and homozyg-window-het 3 (also reported in main text) (two-sided t-test, F_{ROH} ≥100 kb: p = 0.034, F_{ROH} ≥ 2 Mb: p = 0.007); **(c)** homozyg-window-snp 500 and homozyg-window-het 5 (two-sided t-test, F_{ROH} ≥100 kb: p = 0.0274, F_{ROH} ≥ 2 Mb: p = 0.0194); **(d)** ROHan (two-sided t-test, F_{ROH} ≥100 kb: p = 0.0487, F_{ROH} ≥ 2 Mb: p = 0.0434). *n* = 6, *** = p < 0.001, ** = p < 0.01, p-values were not adjusted for multiple comparisons, error bars represent the standard deviation.

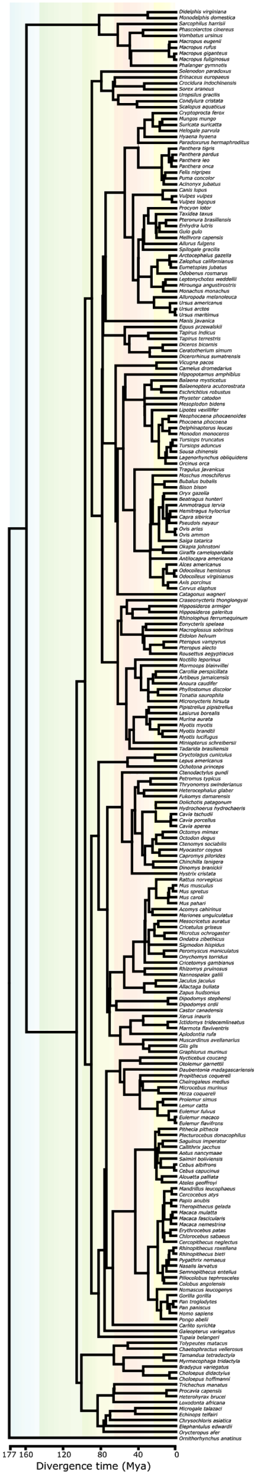

Supplementary Figure 11. **231 mammal genomes used to calculate GERP-scores.**

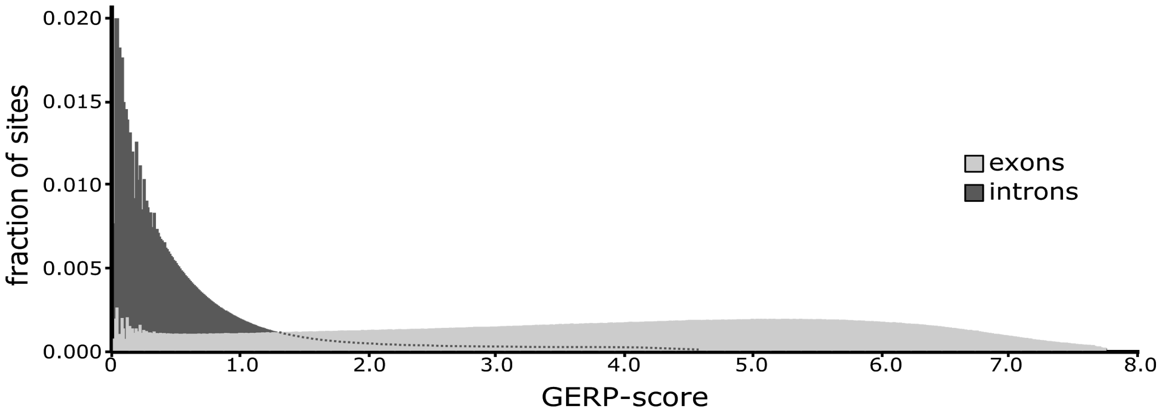

Supplementary Figure 12. Distribution of GERP-scores. Scores are subdivided by non-coding regions (dark grey) and within exons (grey).

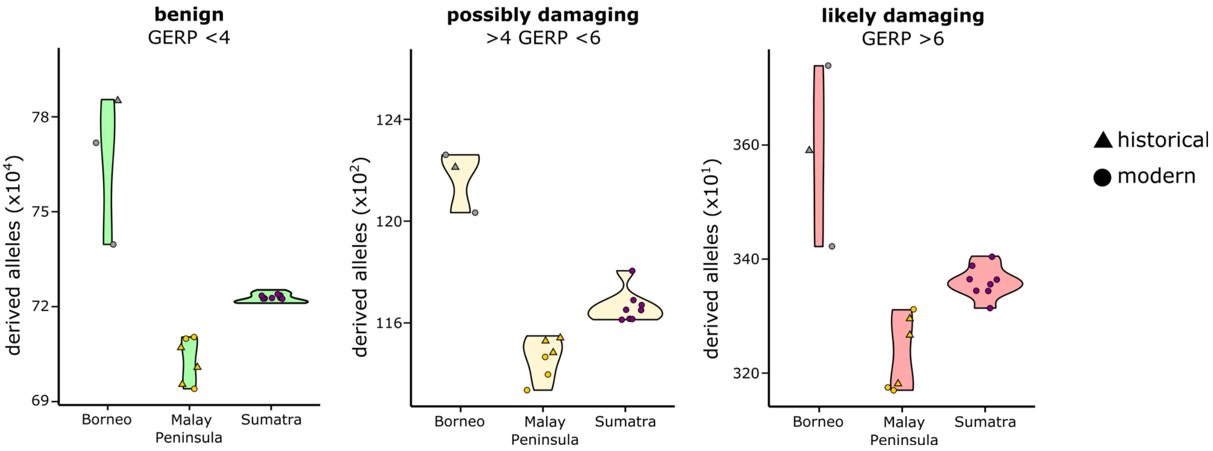

Supplementary Figure 13. Number of derived alleles per individual stratified by GERP-score.

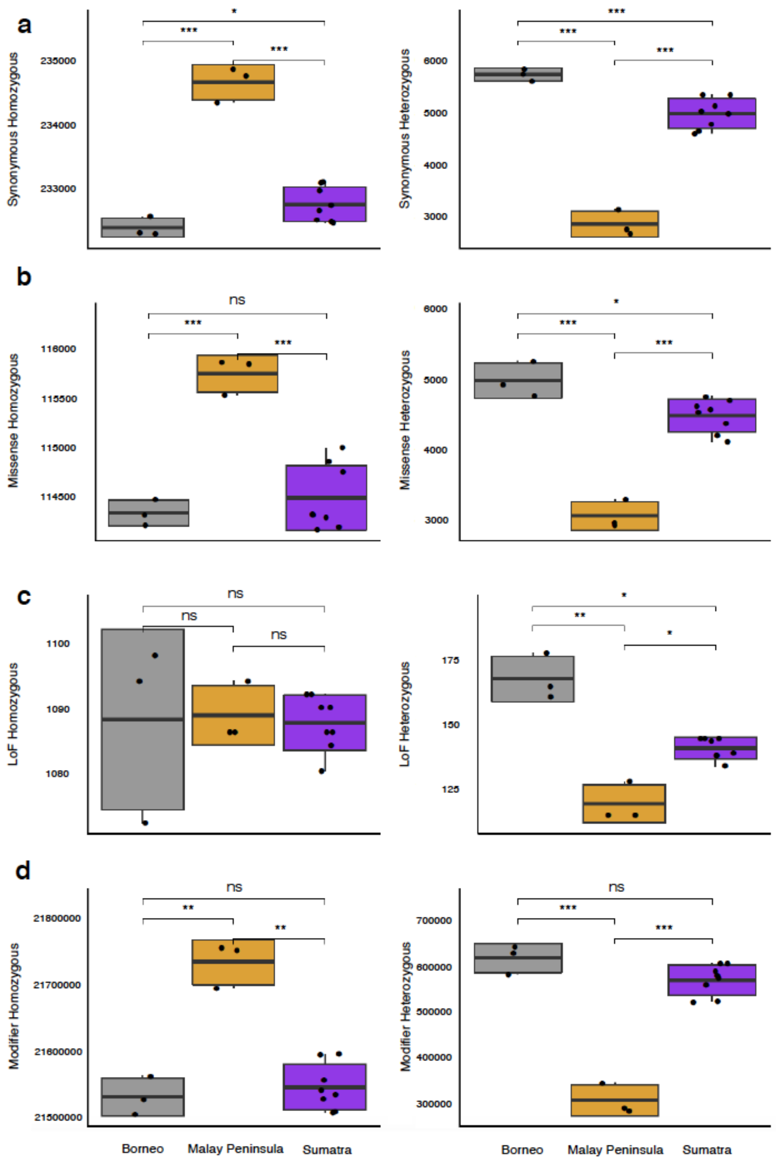

Supplementary Figure 14. Comparison of the number of synonymous and non-synonymous variants within or in proximity of coding regions between modern-day Sumatran rhinoceros populations. The number of variants, separated by homozygous or heterozygous state, in the categories **(a)** Synonymous (two-sided pairwise t-test, homozygous: $p_{Borneo-MalayP}$ = 0.00099, $p_{Borneo-Sumatra}$ = 0.026, $p_{MalayP-Sumatra}$ = 0.00099, heterozygous: $p_{Borneo-MalayP}$ = 5e-04, $p_{Borneo-Sumatra}$ = 0.00022, $p_{MalayP-Sumatra}$ = 0.00022) **(b)** Missense (two-sided pairwise t-test, homozygous: $p_{Borneo-MalayP}$ = 0.00079, $p_{Borneo-Sumatra}$ = 0.3, $p_{MalayP-Sumatra}$ = 0.00013, heterozygous: $p_{Borneo-MalayP}$ = 6e-04, $p_{Borneo-Sumatra}$ = 0.048, $p_{MalayP-Sumatra}$ = 0.00049), **(c)** LoF (two-sided pairwise t-test, homozygous: $p_{Borneo-MalayP}$ = 0.94, $p_{Borneo-Sumatra}$ = 0.96, $p_{MalayP-Sumatra}$ = 0.73, heterozygous: $p_{Borneo-MalayP}$ = 0.0021, $p_{Borneo-Sumatra}$ = 0.026, $p_{MalayP-Sumatra}$ = 0.027) and **(d)** Modifier impact (i.e. downstream or upstream) (two-sided pairwise t-test, homozygous: $p_{Borneo-MalayP}$ = 0.0015, $p_{Borneo-Sumatra}$ = 0.52, $p_{MalayP-Sumatra}$ = 0.0017, heterozygous: $p_{Borneo-MalayP}$ = 0.00031, $p_{Borneo-Sumatra}$ = 0.094, $p_{MalayP-Sumatra}$ = 0.00054). Middle thick line within boxplots and bounds of boxes represent mean and standard deviation, respectively. Vertical lines represent minima and maxima. n = 14, *** = $p < 0.001$, ** = $p < 0.01$, * = $p < 0.05$, ns = non-significant, p-values were not adjusted for multiple comparisons.

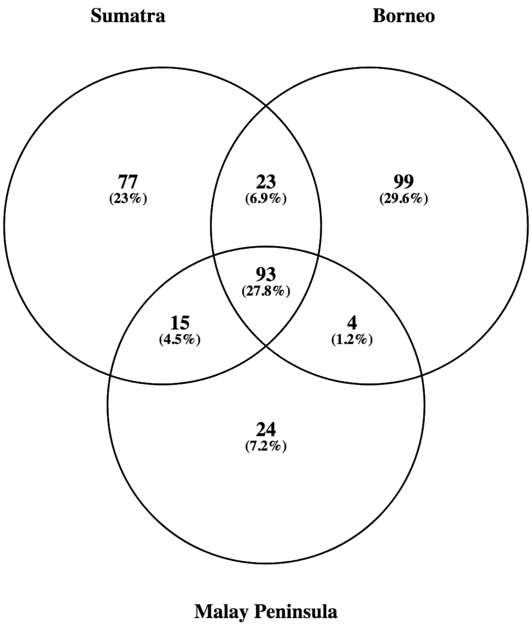

Supplementary Figure 15. Venn diagram for the number of genes with LoF variants in modern populations of Sumatran rhinoceros. Each number corresponds to the number of genes carrying any LoF variant and that are either private to each population or shared between or among populations.

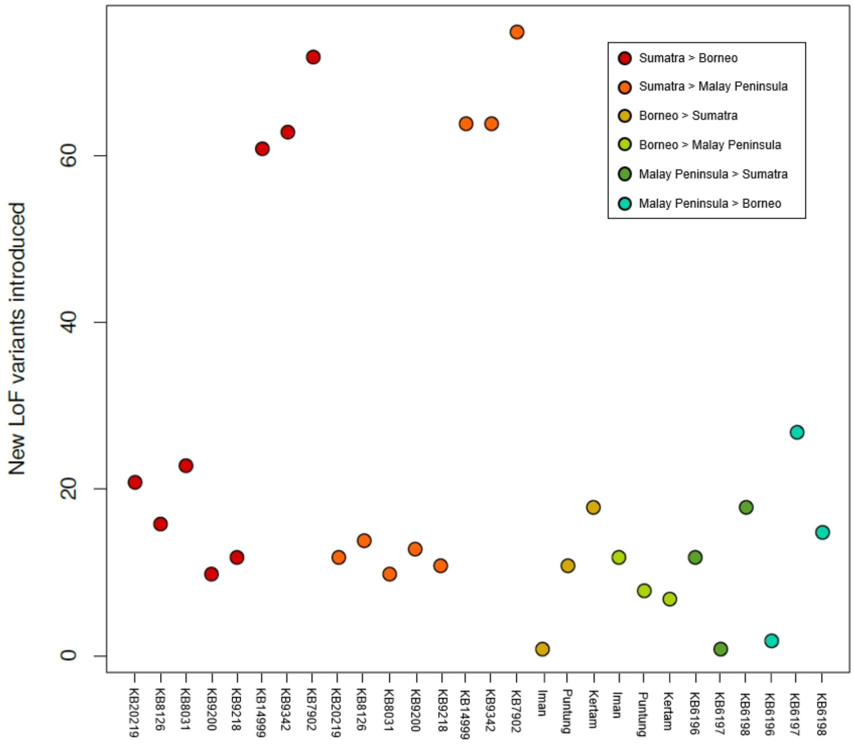

Supplementary Figure 16. Number of new LoF variants introduced by one individual from the first population into the second population if translocation/exchange of gametes would take place.

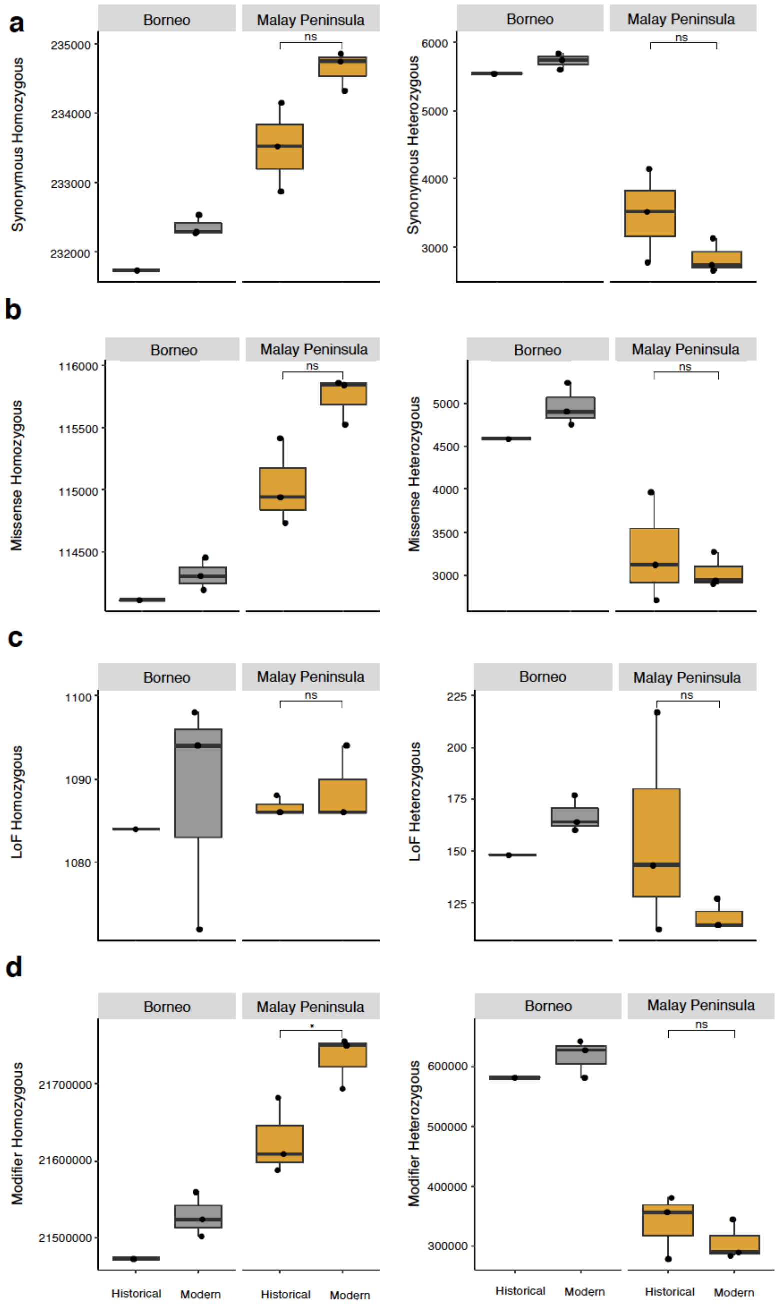

Supplementary Figure 17. **Temporal comparison of the number of synonymous and non-synonymous variants within or in proximity of coding regions between historical and modern genomes in the Bornean and Malay Peninsula populations.** The number of variants, separated by homozygous or heterozygous state, in the categories **(a)** Synonymous **(b)** Missense **(c)** LoF and **(d)** Modifier impact (i.e. downstream or upstream) (two-sided pairwise t-test, homozygous: p = 0.044. Middle thick line within boxplots and bounds of boxes represent mean and standard deviation, respectively. Vertical lines represent minima and maxima. n = 6, * = p <0.05, ns = non-significant, p-values were not adjusted for multiple comparisons.

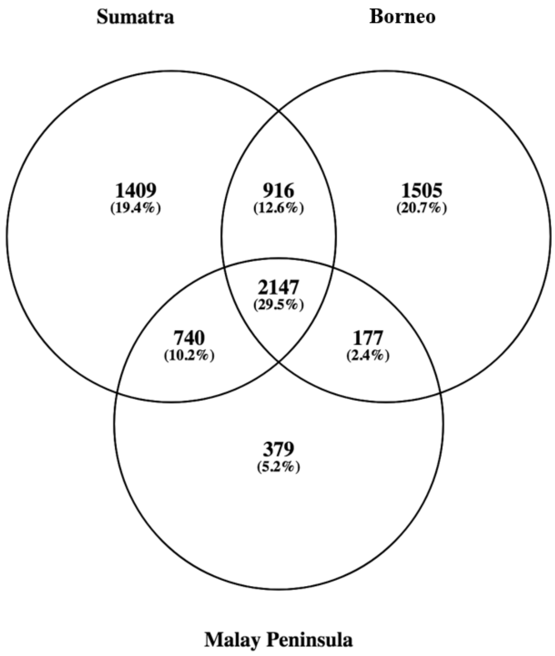

Supplementary Figure 18. **Venn diagram for the number of genes with missense variants in modern populations of Sumatran rhinoceros.** Each number corresponds to the number of genes carrying any missense variant and that are either private to each population or shared between or among populations.

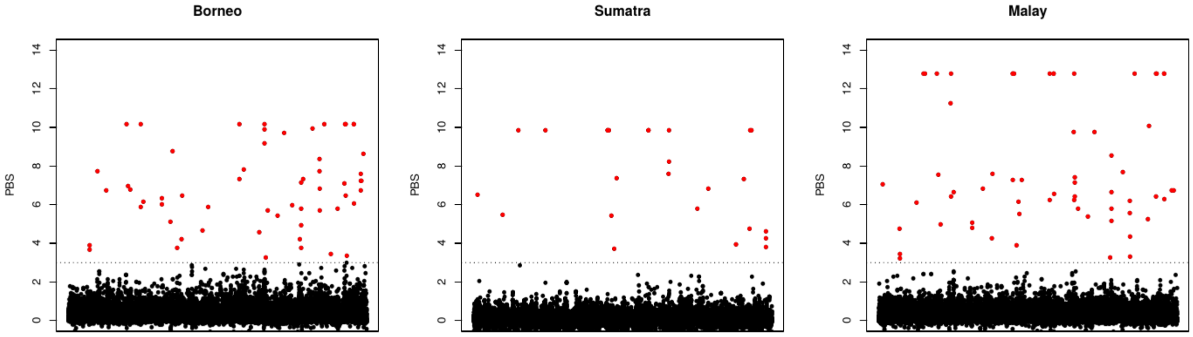

Supplementary Figure 19. PBS score for all gene models in three Sumatran rhinoceros populations. Red dots represent genes with PBS score larger than 3 (see text), candidates of being under positive selection in that population. In order to depict all outliers, genes with infinite PBS score were replaced by the maximum value of the distribution of PBS scores for that population.

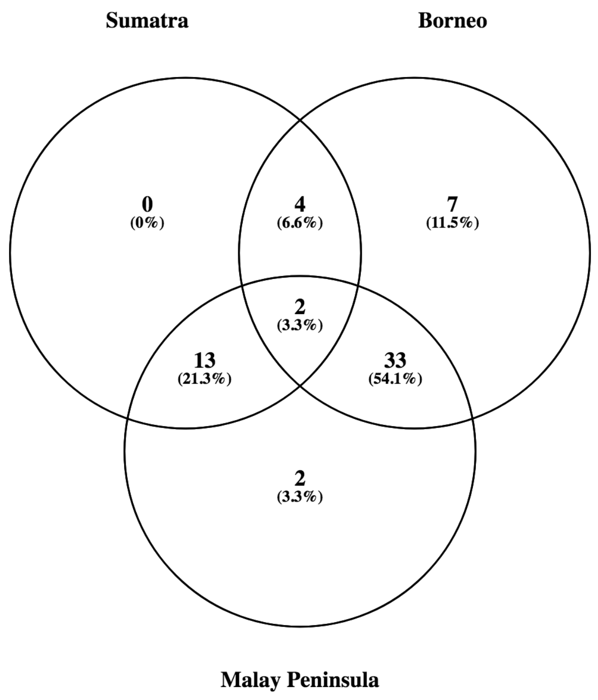

Supplementary Figure 20. Venn diagram for genes under putative positive selection identified with the PBS in modern populations of Sumatran rhinoceros.

Supplementary tables

Supplementary Table 1. Sample list historical and modern Sumatran rhinoceros genomes. The list includes the five historical and 16 modern specimens that were re-sequenced as well as all other museum specimens screened for endogenous DNA content.

Sample ID	*Museum institution*	*Museum ID*	*Sampling location*	*Region*	*Collection date*	*Time period*	*Type of tissue*	*Remark*
SR01	NHM London	1886.12.20.8	N. Borneo	Borneo	1886	Historical	Tooth	
SR08	NHM London	1921.2.83	Ulu Benus, Pehony, Malay Peninsular	Malay Peninsula	1921	Historical	Tooth	
SR09	NHM London	1921.2.8.2	Ulu Benus, Pehony, Malay Peninsular	Malay Peninsula	5/16/1916	Historical	Tooth	
SR12	NHM London	1875.8.9.18	Borneo	Borneo	1875	Historical	Petrous bone	
SR22	RBINS Brussels	RBINS1204	Sumatra coast	Malay Peninsula	5/15/1879	Historical	Tooth	probably mislabelled; clustering with Malay Peninsula
Gelugob			Kinabatangan	Sabah, Borneo		Modern	Soft tissue	
Iman			Danum Valley Conservation Area	Sabah, Borneo		Modern	Blood	
KB14999			NY Bronx Zoo, USA	Sumatra		Modern	Fibroblast cells	
KB20219			Cincinnati Zoo, USA	Captive born, parents from Sumatra		Modern	Skin	captive born, offspring of KB7902 and KB9342
KB6196			Melaka Zoo, Malaysia	Malay Peninsula		Modern	Fibroblast cells	
KB6197			Melaka Zoo, Malaysia	Malay Peninsula		Modern	Fibroblast cells	
KB6198			Melaka Zoo, Malaysia	Malay Peninsula		Modern	Fibroblast cells	
KB7902			San Diego Zoo, USA	Bengkulu, Sumatra		Modern	Muscle	father to KB20219
KB8031			San Diego Zoo, USA	Bengkulu, Sumatra		Modern	Heart	
KB8126			Cincinnati Zoo, USA	Riau province, Sumatra		Modern	Heart	
KB9200			San Diego Zoo, USA	Kumu river, Riau province, Sumatra		Modern	Heart	
KB9218			San Diego Zoo, USA	Dunga Tajung, Sumatra		Modern	Heart	
KB9342			LA Zoo/Cincinnati Zoo, USA	Kerinci Seblat National Park, Sumatra		Modern	Fibroblast cells	mother to KB20219
Kertam			Kretam Forest Reserve	Sabah, Borneo		Modern	Blood	
OR2142			SOS RHINO, Malaysia	Borneo		Modern	Skin	
Puntung			Tabin Wildlife Reserve	Sabah, Borneo		Modern	Blood	
SR02	NHM London	2004.23	Sumatra	Indonesia	1980 earliest	Historical	Ulna	Screened for endogenous DNA content only
SR03	NHM London	1894.9.24.1	Sumatra	Indonesia	1894	Historical	Tooth	Not screened
SR04	NHM London	1949.1.11.1	NA	NA	1949	Historical	Petrous bone	Screened for endogenous DNA content only
SR05	NHM London	1879.6.14.2	Mount Ophir, Malay Peninsular	Malaysia	1879	Historical	Tooth	Screened for endogenous DNA content only
SR06	NHM London	1931.5.28.1		Burma	1931	Historical	Tooth/Bone	Screened for endogenous DNA content only
SR07	NHM London	1921.2.8.1	Ulu Kenabor, Negu, Sembelam, Malay penisular	Malaysia	1921	Historical	Tooth	Screened for endogenous DNA content only
SR10	NHM London	1921.2.8.4	Ulu Benus, Pehony, Malay Peninsular	Malaysia	1921	Historical	Tooth	Screened for endogenous DNA content only
SR11	NHM London	1868.4.15.1, 1461.a	Pegu Burma	Burma	1868	Historical	Tooth	Screened for endogenous DNA content only
SR13	NHM London	1879.3.11.1	Sagalint Sandakam, NE Borneo	Malaysia	1879	Historical	Tooth	Screened for endogenous DNA content only
SR14	NHM London	1952.4.1.2	Sumatra	Indonesia	1952	Historical	Tooth	Screened for endogenous DNA content only
SR15	NHM London	1972.72, 1461.b	Sumatra?	Indonesia	1972	Historical	Tooth	Screened for endogenous DNA content only
SR16	NHM London	1948.1.14.1		NA	1948	Historical	Tooth	Screened for endogenous DNA content only
SR17	NHM London	1949.2.1.1		NA	1949	Historical	Tooth	Screened for endogenous DNA content only
SR18	NHM London	1950.3.16.1		NA	1950	Historical	Base of horn	Screened for endogenous DNA content only
SR19	NHM Copenhagen	CN 3791	Sumatra	Indonesia	1959 earliest	Historical	Tooth	Screened for endogenous DNA content only
SR20	NHM Copenhagen	CN 617	Sumatra	Indonesia	1893	Historical	Tooth	Screened for endogenous DNA content only
SR21	RBINS Brussels	RBINS 1203	Sumatra	Indonesia	1930	Historical	Tooth	Screened for endogenous DNA content only
SR23	NRM Stockholm	NRM601571	Java	Indonesia	1886	Historical	Tooth	Screened for endogenous DNA content only
SR24	NMW Vienna	NMW 1500	NA	NA	1884	Historical	Tooth	Screened for endogenous DNA content only
SR25	NMW Vienna	NMW 3082	NA	NA	11/10/1910	Historical	Tooth	Screened for endogenous DNA content only
SR26	NMW Vienna	NMW 4294	NA	NA	1873	Historical	Tooth	Screened for endogenous DNA content only
SR27	NMW Vienna	NMW 7529	NA	NA	9/16/1920	Historical	Tooth	Screened for endogenous DNA content only
SR28	NMW Vienna	NMW 8173	Upper Mekong	Laos	1907	Historical	Bone	Screened for endogenous DNA content only
SR29	NMW Vienna	NMW 29566	Sumatra	Indonesia	1980	Historical	Petrous bone	Screened for endogenous DNA content only
SR30	NMW Vienna	NMW 29567	Sumatra	Indonesia		Historical	Jaw bone	Screened for endogenous DNA content only
SR31	NMW Vienna	NMW 29568	NA	Indonesia		Historical	Long bone	Screened for endogenous DNA content only

Supplementary Table 2. Read statistics for historical and modern Sumatran rhinoceros genomes. The list includes the five historical and 16 modern specimens that were re-sequenced as well as all other museum specimens screened for endogenous DNA content.

Sample ID	*Average coverage*	*N reads*	*Mapping quality*	*Endogenous DNA content (%)*	*Remark*
SR01	3	110 733 499	24.86	36.6	
SR08	9	242 363 739	28.72	52.6	
SR09	11	359 191 060	27.47	71.1	
SR12	13	434 847 132	28.02	72.4	
SR22	10	546 873 284	25.46	88.7	
Gelugob	2	127 879 241	41.31	NA	
Iman	29	606 237 054	48.43	NA	
KB14999	21	412 106 534	48.05	NA	
KB20219	20	392 563 239	48.09	NA	
KB6196	18	343 617 954	48.18	NA	
KB6197	17	315 915 276	48.13	NA	
KB6198	20	383 911 880	47.98	NA	
KB7902	20	375 108 971	48.78	NA	
KB8031	21	400 329 549	48.09	NA	
KB8126	22	418 453 049	47.93	NA	
KB9200	21	397 326 810	47.92	NA	
KB9218	19	370 893 451	48.51	NA	
KB9342	22	428 656 994	48.17	NA	
Kertam	21	444 726 728	50.48	NA	
OR2142	5	316 088 180	44.23	NA	
Puntung	19	378 891 330	48.48	NA	
SR02	NA	NA	NA	62.8	Screened for endogenous DNA content only
SR04	NA	NA	NA	16.7	Screened for endogenous DNA content only
SR05	NA	NA	NA	61.0	Screened for endogenous DNA content only
SR06	NA	NA	NA	56.0	Screened for endogenous DNA content only
SR07	NA	NA	NA	3.0	Screened for endogenous DNA content only
SR10	NA	NA	NA	48.3	Screened for endogenous DNA content only
SR11	NA	NA	NA	83.3	Screened for endogenous DNA content only
SR13	NA	NA	NA	25.0	Screened for endogenous DNA content only
SR14	NA	NA	NA	49.7	Screened for endogenous DNA content only
SR15	NA	NA	NA	65.6	Screened for endogenous DNA content only
SR16	NA	NA	NA	67.7	Screened for endogenous DNA content only
SR17	NA	NA	NA	44.3	Screened for endogenous DNA content only
SR18	NA	NA	NA	2.8	Screened for endogenous DNA content only
SR19	NA	NA	NA	2.7	Screened for endogenous DNA content only
SR20	NA	NA	NA	6.8	Screened for endogenous DNA content only
SR21	NA	NA	NA	52.8	Screened for endogenous DNA content only
SR23	NA	NA	NA	1.4	Screened for endogenous DNA content only
SR24	NA	NA	NA	4.6	Screened for endogenous DNA content only
SR25	NA	NA	NA	10.0	Screened for endogenous DNA content only
SR26	NA	NA	NA	18.5	Screened for endogenous DNA content only
SR27	NA	NA	NA	4.1	Screened for endogenous DNA content only
SR28	NA	NA	NA	25.9	Screened for endogenous DNA content only
SR29	NA	NA	NA	41.9	Screened for endogenous DNA content only
SR30	NA	NA	NA	1.3	Screened for endogenous DNA content only
SR31	NA	NA	NA	2.1	Screened for endogenous DNA content only

Supplementary Table 3. Pairwise F_{ST} values between Sumatra, Malay Peninsula and Borneo Sumatran rhinoceros populations.

	Sumatra	*Malay Peninsula*	*Borneo*
Sumatra	0		
Malay Peninsula	0.265	0	
Borneo	0.343	0.267	0

Supplementary Table 4. Divergence time estimates between Sumatran rhinoceros populations using the PSMC approach.

Population comparison	*N discrete intervals*	*Start of divergence (years BP)*
Borneo - Sumatra	64	29,893
	49	35,123
	37	41,043
Borneo - Malay Peninsula	64	30,809
	49	25,831
	37	30,114
Sumatra - Malay Peninsula	64	9,412
	49	13,479
	37	11,657

Supplementary Table 5. Mean F_{ROH} of the populations on Sumatra, modern and historical Borneo, as well as modern and historical Malay Peninsula. $^{*}F_{ROH}$ was estimated for one sample only for historical Borneo.

Population	*Mean $F_{ROH} \geq 100$ kb*	*Mean $F_{ROH} \geq 2$ Mb*
Modern Sumatra	0.31	0.086
Modern Borneo	0.21	0.045
Historical Borneo*	0.14	0.019
Modern Malay Peninsula	0.65	0.30
Historical Malay Peninsula	0.44	0.078

Supplementary Table 6. **Percentage of derived alleles for mutational load estimated using GERP-scores (GERP-score > 4) unique to each population or shared between populations.**

Population	*Unique/shared derived alleles (%)*
Borneo	30.1
Sumatra	19.9
Malay Peninsula	14.1
Borneo - Sumatra	5.1
Borneo - Malay Peninsula	3.1
Sumatra - Malay Peninsula	10.1
Borneo - Sumatra - Malay Peninsula	17.6

Supplementary Table 7. Overall and per population total number of LoF variants and genes carrying LoF variants. For each population, the total number of private, fixed, private + fixed LoF variants, as well as the total number of private genes carrying LoF variants is also listed.

Overall			
LoF variants		373	
Genes with LoF variants		335	
Per population	*Sumatra*	*Borneo*	*Malay Peninsula*
LoF variants	233	245	154
Private LoF variants	86	109	25
Fixed LoF variants	0	7	13
Private and fixed LoF variants	0	2	0
Private genes with LoF variants	77	99	24

Supplementary Table 8. Number of genes with at least one LoF variant for the three modern Sumatran rhinoceros populations.

Number of LoF variants per gene	*Sumatra*	*Borneo*	*Malay Peninsula*
1	191	198	123
2	12	16	8
3	4	5	5
4	0	0	0
5	0	0	0
6	1	0	0

Supplementary Table 9. Gene function analysis for LoF variants.

Biological process	*N genes*	*Percent of gene hit against total number of genes*	*Percent of gene hit against total number of Pathway hits*
cellular process (GO:0009987)	74	34.60%	28.00%
metabolic process (GO:0008152)	50	23.40%	18.90%
biological regulation (GO:0065007)	41	19.20%	15.50%
response to stimulus (GO:0050896)	21	9.80%	8.00%
signaling (GO:0023052)	19	8.90%	7.20%
cellular component organization or biogenesis (GO:0071840)	17	7.90%	6.40%
localization (GO:0051179)	15	7.00%	5.70%
developmental process (GO:0032502)	10	4.70%	3.80%
multicellular organismal process (GO:0032501)	8	3.70%	3.00%
immune system process (GO:0002376)	4	1.90%	1.50%
multi-organism process (GO:0051704)	2	0.90%	0.80%
reproductive process (GO:0022414)	1	0.50%	0.40%
reproduction (GO:0000003)	1	0.50%	0.40%
biological adhesion (GO:0022610)	1	0.50%	0.40%

Supplementary Table 10. Overall and per population total number of missense variants and genes with missense variants. For each population, the total number of private, as well as private + fixed genes carrying missense variants is also listed. Missense variants that were fixed in all three populations are excluded.

Overall			
Missense variants		15,598	
Genes with missense variants		6,490	
Per population	*Sumatra*	*Borneo*	*Malay Peninsula*
Private genes with missense variants	1,409	1,505	379
Private and fixed genes with missense variants	0	103	107

Supplementary Table 11. Summary of PBS analysis. Number of high quality gene models, number of genes with PBS values above the 99.8th percentile of the distribution of the PBS values of all genes, number of genes with an infinite PBS value (i.e., F_{ST} = 1) over all pairwise population comparisons, number of genes with a signal for positive selection in common with missense analysis (Supplementary Table 10), and number of genes with a signal for positive selection unique to each population.

Overall			
High quality gene models		33,026	
Genes > 99.8th percentile		61	
Genes F_{ST} = 1		12	
Genes in common with missense analysis		2	
Per population	*Sumatra*	*Borneo*	*Malay Peninsula*
Private genes with signal for positive selection	0	7	2

Supplementary Table 12. Gene function analysis for (a) missense variants and (b) genes under positive selection (PBS approach) for the three modern Sumatran rhinoceros populations.

(a) Missense variants

	Biological process	*N genes*	*Percent of gene hit against total number of genes*	*Percent of gene hit against total number of Pathway hits*
Sumatra	cellular process (GO:0009987)	110	36.5%	26.6%
	metabolic process (GO:0008152)	55	18.3%	13.3%
	cellular component organization or biogenesis (GO:0071840)	52	17.3%	12.6%
	biological regulation (GO:0065007)	50	16.6%	12.1%
	localization (GO:0051179)	36	12.0%	8.7%
	response to stimulus (GO:0050896)	31	10.3%	7.5%
	multicellular organismal process (GO:0032501)	22	7.3%	5.3%
	developmental process (GO:0032502)	17	5.6%	4.1%
	signaling (GO:0023052)	16	5.3%	3.9%
	locomotion (GO:0040011)	7	2.3%	1.7%
	reproduction (GO:0000003)	3	1.0%	0.7%
	reproductive process (GO:0022414)	3	1.0%	0.7%
	biological adhesion (GO:0022610)	3	1.0%	0.7%
	immune system process (GO:0002376)	3	1.0%	0.7%
	biological phase (GO:0044848)	3	1.0%	0.7%
	multi-organism process (GO:0051704)	1	0.3%	0.2%
	growth (GO:0040007)	1	0.3%	0.2%
	pigmentation (GO:0043473)	1	0.3%	0.2%

Borneo	cellular process (GO:0009987)	108	36.0%	23.9%
	biological regulation (GO:0065007)	63	21.0%	14.0%
	metabolic process (GO:0008152)	57	19.0%	12.6%
	cellular component organization or biogenesis (GO:0071840)	53	17.7%	11.8%
	localization (GO:0051179)	38	12.7%	8.4%
	response to stimulus (GO:0050896)	34	11.3%	7.5%
	signaling (GO:0023052)	23	7.7%	5.1%
	multicellular organismal process (GO:0032501)	20	6.7%	4.4%
	developmental process (GO:0032502)	19	6.3%	4.2%
	locomotion (GO:0040011)	10	3.3%	2.2%
	immune system process (GO:0002376)	7	2.3%	1.6%
	biological adhesion (GO:0022610)	6	2.0%	1.3%
	multi-organism process (GO:0051704)	4	1.3%	0.9%
	reproduction (GO:0000003)	3	1.0%	0.7%
	reproductive process (GO:0022414)	3	1.0%	0.7%
	growth (GO:0040007)	1	0.3%	0.2%
	pigmentation (GO:0043473)	1	0.3%	0.2%
	biological phase (GO:0044848)	1	0.3%	0.2%

Malay Peninsula	cellular process (GO:0009987)	102	34.6%	23.6%
	cellular component organization or biogenesis (GO:0071840)	56	19.0%	12.9%
	biological regulation (GO:0065007)	56	19.0%	12.9%
	metabolic process (GO:0008152)	46	15.6%	10.6%
	localization (GO:0051179)	42	14.2%	9.7%
	response to stimulus (GO:0050896)	32	10.8%	7.4%
	signaling (GO:0023052)	22	7.5%	5.1%
	developmental process (GO:0032502)	21	7.1%	4.8%
	multicellular organismal process (GO:0032501)	21	7.1%	4.8%
	locomotion (GO:0040011)	11	3.7%	2.5%
	immune system process (GO:0002376)	6	2.0%	1.4%
	multi-organism process (GO:0051704)	4	1.4%	0.9%
	biological adhesion (GO:0022610)	4	1.4%	0.9%
	reproduction (GO:0000003)	3	1.0%	0.7%
	reproductive process (GO:0022414)	3	1.0%	0.7%
	growth (GO:0040007)	2	0.7%	0.5%
	cell population proliferation (GO:0008283)	1	0.3%	0.2%
	biological phase (GO:0044848)	1	0.3%	0.2%

(b) Genes under positive selection (PBS)

	Biological process	*N genes*	*Percent of gene hit against total number of genes*	*Percent of gene hit against total number of Pathway hits*
Sumatra	cellular component organization or biogenesis (GO:0071840)	1	7.1%	4.5%
	cellular process (GO:0009987)	4	28.6%	18.2%
	multi-organism process (GO:0051704)	1	7.1%	4.5%
	localization (GO:0051179)	3	21.4%	13.6%
	biological regulation (GO:0065007)	3	21.4%	13.6%
	response to stimulus (GO:0050896)	3	21.4%	13.6%
	signaling (GO:0023052)	3	21.4%	13.6%
	locomotion (GO:0040011)	1	7.1%	4.5%
	metabolic process (GO:0008152)	2	14.3%	9.1%
	immune system process (GO:0002376)	1	7.1%	4.5%
Borneo	cellular component organization or biogenesis (GO:0071840)	4	12.5%	7.7%
	cellular process (GO:0009987)	17	53.1%	32.7%
	localization (GO:0051179)	2	6.3%	3.8%
	biological regulation (GO:0065007)	8	25.0%	15.4%
	response to stimulus (GO:0050896)	3	9.4%	5.8%
	signaling (GO:0023052)	3	9.4%	5.8%
	developmental process (GO:0032502)	2	6.3%	3.8%
	multicellular organismal process (GO:0032501)	2	6.3%	3.8%
	metabolic process (GO:0008152)	10	31.3%	19.2%
	cell population proliferation (GO:0008283)	1	3.1%	1.9%
Malay Peninsula	cellular component organization or biogenesis (GO:0071840)	6	16.7%	10.3%
	cellular process (GO:0009987)	16	44.4%	27.6%
	multi-organism process (GO:0051704)	1	2.8%	1.7%
	localization (GO:0051179)	6	16.7%	10.3%
	biological regulation (GO:0065007)	7	19.4%	12.1%
	response to stimulus (GO:0050896)	2	5.6%	3.4%
	signaling (GO:0023052)	3	8.3%	5.2%
	developmental process (GO:0032502)	2	5.6%	3.4%
	multicellular organismal process (GO:0032501)	2	5.6%	3.4%
	locomotion (GO:0040011)	1	2.8%	1.7%
	metabolic process (GO:0008152)	10	27.8%	17.2%
	cell population proliferation (GO:0008283)	1	2.8%	1.7%
	immune system process (GO:0002376)	1	2.8%	1.7%

Supplementary note 1: TimeTree generated dated phylogeny used as input for GERP++

((((Didelphis_virginiana:30.00000000,Monodelphis_domestica:30.00000000)'14':51.50867481,(Sarcophilus_ha
rrisii:61.62901000,((Phascolarctos_cinereus:35.00972500,Vombatus_ursinus:35.00972500)'13':13.93526577,(((
Macropus_eugenii:6.80755333,Macropus_rufus:6.80755333)'11':0.50744667,(Macropus_giganteus:1.97250000
,Macropus_fuliginosus:1.97250000)'10':5.34250000)'19':40.28056667,Phalanger_gymnotis:47.59556667)'9':1.3
4942410)'22':12.68401923)'8':19.87966481)'6':77.08891276,((((Solenodon_paradoxus:79.27050000,((Erinaceus
_europaeus:64.77156154,(Crocidura_indochinensis:33.74066667,Sorex_araneus:33.74066667)'30':31.03089487
)'29':1.99932937,(Uropsilus_gracilis:61.30000000,(Condylura_cristata:41.20000000,Scalopus_aquaticus:41.200
00000)'27':20.10000000)'35':5.47089091)'43':12.49960909)'42':10.05331840,(((((((((Cryptoprocta_ferox:24.556
35936,((Mungos_mungo:14.49250000,Suricata_suricatta:14.49250000)'40':2.60750000,Helogale_parvula:17.10
000000)'48':7.45635936)'51':8.84757492,Hyaena_hyaena:33.40393429)'47':6.49606571,Paradoxurus_hermaphr
oditus:39.90000000)'39':0.00000000,((Panthera_tigris:7.41234111,((Panthera_pardus:3.82497571,Panthera_leo:
3.82497571)'56':0.87983629,Panthera_onca:4.70481200)'55':2.70752911)'61':7.76265306,((Felis_nigripes:11.52
134800,Puma_concolor:11.52134800)'60':1.47865200,Acinonyx_jubatus:13.00000000)'54':2.17499417)'38':24.
72500583)'34':14.42144118,(((Lycaon_pictus:7.80599657,Canis_lupus:7.80599657)'26':6.34729293,(Vulpes_vu
lpes:4.22904857,Vulpes_lagopus:4.22904857)'66':9.92424093)'75':31.37506017,(((((Procyon_lotor:29.3054353
3,(Taxidea_taxus:21.11916583,(((Pteronura_brasiliensis:10.62861800,Enhydra_lutris:10.62861800)'80':6.87138
200,Gulo_gulo:17.50000000)'78':3.05000500,Mellivora_capensis:20.55000500)'74':0.56916083)'83':8.1862695
0)'73':4.67159967,Ailurus_fulgens:33.97703500)'88':0.97040000,Spilogale_gracilis:34.94743500)'86':4.879188
08,(((Arctocephalus_gazella:6.90256862,(Zalophus_californianus:5.65553150,Eumetopias_jubatus:5.65553150
)'72':1.24703713)'93':12.56705445,Odobenus_rosmarus:19.46962308)'92':6.51564835,((Leptonychotes_weddell
ii:11.07048875,Mirounga_angustirostris:11.07048875)'91':2.39709569,Monachus_monachus:13.46758444)'71':
12.51768698)'69':13.84135165)'65':0.06678959,(Ailuropoda_melanoleuca:23.36263333,(Ursus_americanus:6.4
4007273,(Ursus_arctos:1.08895385,Ursus_maritimus:1.08895385)'25':5.35111888)'5':16.92256061)'102':16.530
77933)'100':5.63493700)'107':8.79309151)'111':20.33882610,Manis_javanica:74.66026727)'110':2.56632673,((
Equus_przewalskii:7.72000000,Equus_asinus:7.72000000)'106':46.68072903,((Tapirus_indicus:29.30000000,T
apirus_terrestris:29.30000000)'129':20.26268000,((Diceros_bicornis:14.12077500,Ceratotherium_simum:14.12
077500)'128':13.80092500,Dicerorhinus_sumatrensis:27.92170000)'133':21.64098000)'127':4.83804903)'136':2
2.82586497)'126':0.52836241,((Vicugna_pacos:20.56159000,Camelus_dromedarius:20.56159000)'142':43.6224
3407,(((Hippopotamus_amphibius:53.75023500,((Balaena_mysticetus:25.90486500,(Balaenoptera_acutorostrat
a:15.53758200,Eschrichtius_robustus:15.53758200)'146':10.36728300)'150':7.59513500,(Physeter_catodon:33.
50000000,(Mesoplodon_bidens:33.03879294,(Lipotes_vexillifer:25.41895647,(((Neophocaena_phocaenoides:7
.59597125,Phocoena_phocoena:7.59597125)'149':6.69456086,(Delphinapterus_leucas:6.97528600,Monodon_m
onoceros:6.97528600)'145':7.31524611)'141':4.10074167,((((Tursiops_truncatus:3.59849364,Tursiops_aduncus:
3.59849364)'140':2.08150636,Sousa_chinensis:5.68000000)'139':4.23000000,Lagenorhynchus_obliquidens:9.9
1000000)'125':1.23236857,Orcinus_orca:11.14236857)'124':7.24890521)'160':7.02768269)'159':7.61983647)'15
8':0.46120706)'157':0.00000000)'123':20.25023500)'122':2.20961810,(Tragulus_javanicus:43.96862857,((((Mos
chus_moschiferus:24.60000000,((Bubalus_bubalis:12.28692556,Bison_bison:12.28692556)'121':12.31307444,(
((Oryx_gazella:12.72799143,Beatragus_hunteri:12.72799143)'120':1.10400720,((Ammotragus_lervia:9.750000
00,((Hemitragus_hylocrius:6.11000000,Capra_sibirica:6.11000000)'119':0.91535250,Pseudois_nayaur:7.025352
50)'118':2.72464750)'117':0.00000000,(Ovis_aries:0.95980625,Ovis_ammon:0.95980625)'115':8.79019375)'105
':4.08199862)'99':4.16800138,Saiga_tatarica:18.00000000)'177':6.60000000)'185':0.00000000)'184':2.07359250
,(Okapia_johnstoni:13.81482500,Giraffa_camelopardalis:13.81482500)'183':12.85876750)'182':0.49277150,Ant
ilocapra_americana:27.16636400)'180':0.14091700,((Alces_americanus:9.80990000,((Odocoileus_hemionus:1.
97500000,Odocoileus_virginianus:1.97500000)'176':5.32930000,Rangifer_tarandus:7.30430000)'192':2.505600
00)'195':3.79010000,(Axis_porcinus:8.53958333,Cervus_elaphus:8.53958333)'191':5.06041667)'175':13.70728
100)'205':16.66134757)'204':11.99122453)'203':6.00613542,Catagonus_wagneri:61.96598852)'202':2.21803556
)'201':13.57093234)'212':0.77378567,(((Craseonycteris_thonglongyai:52.22052625,((Hipposideros_armiger:28.
40000000,Hipposideros_galeritus:28.40000000)'200':17.70000000,Rhinolophus_ferrumequinum:46.10000000)'

218':6.12052625)'229':5.85794851,((Eonycteris_spelaea:31.79261000,Macroglossus_sobrinus:31.79261000)'22
8':3.40739000,(Eidolon_helvum:33.75681333,((Pteropus_vampyrus:12.94490000,Pteropus_alecto:12.94490000
)'227':14.43589692,Rousettus_aegyptiacus:27.38079692)'226':6.37601641)'225':1.44318667)'224':22.87847476
)'223':3.58273310,((Noctilio_leporinus:43.04001263,(Mormoops_blainvillei:37.05923789,(((Carollia_perspicill
ata:21.50000000,Artibeus_jamaicensis:21.50000000)'243':2.89934186,Anoura_caudifer:24.39934186)'242':5.00
065814,((Phyllostomus_discolor:19.13619545,Tonatia_saurophila:19.13619545)'241':10.26380455,Micronycter
is_hirsuta:29.40000000)'240':0.00000000)'239':7.65923789)'251':5.98077474)'250':9.60601087,((((Pipistrellus_
pipistrellus:29.00000000,Lasiurus_borealis:29.00000000)'238':2.32232500,(Murina_aurata:27.47313125,(Myoti
s_myotis:18.14394667,(Myotis_brandtii:14.19174800,Myotis_lucifugus:14.19174800)'222':3.95219867)'221':9.
32918458)'217':3.84919375)'216':13.51340429,Miniopterus_schreibersii:44.83572929)'265':4.36427071,Tadari
da_brasiliensis:49.20000000)'268':3.44602350)'264':9.01518436)'274':16.86753423)'273':10.79507632)'272':7.1
3857077,((((Oryctolagus_cuniculus:22.18401706,Lepus_americanus:22.18401706)'271':29.24433394,Ochotona
_princeps:51.42835100)'263':30.71244789,((Ctenodactylus_gundi:57.10000000,((((Petromus_typicus:26.93785
600,Thryonomys_swinderianus:26.93785600)'262':13.17842971,(Heterocephalus_glaber:33.83285900,Fukomy
s_damarensis:33.83285900)'261':6.28342671)'260':3.24218720,(((Dolichotis_patagonum:21.50000000,Hydroch
oerus_hydrochaeris:21.50000000)'259':0.00000000,((Cavia_tschudii:5.42431500,Cavia_porcellus:5.42431500)'
257':0.23247500,Cavia_aperea:5.65679000)'215':15.84321000)'199':14.27899950,((((Octomys_mimax:14.0000
0000,Octodon_degus:14.00000000)'284':6.83643601,Ctenomys_sociabilis:20.83643601)'198':4.04398921,(Myo
castor_coypus:17.58582415,Capromys_pilorides:17.58582415)'174':7.29460107)'173':7.97493944,(Chinchilla_l
anigera:24.06609769,Dinomys_branickii:24.06609769)'298':8.78926697)'296':2.92363483)'301':7.57947342)'2
95':3.17190486,Hystrix_cristata:46.53037778)'305':10.56962222)'308':15.77670816,((((((((Rattus_norvegicus:2
0.88741740,(((Mus_musculus:3.06548222,Mus_spretus:3.06548222)'304':4.34711578,Mus_caroli:7.41259800)'
294':0.87822836,Mus_pahari:8.29082636)'313':12.59659104)'317':7.68810347,(Acomys_cahirinus:24.8965460
6,Meriones_unguiculatus:24.89654606)'316':3.67897481)'312':4.08810639,((((Mesocricetus_auratus:18.700000
00,Cricetulus_griseus:18.70000000)'327':4.12601462,(Microtus_ochrogaster:12.07929111,Ondatra_zibethicus:1
2.07929111)'326':10.74672350)'332':5.97398538,Sigmodon_hispidus:28.80000000)'331':0.00000000,(Peromysc
us_maniculatus:12.74010167,Onychomys_torridus:12.74010167)'325':16.05989833)'324':3.86362726)'323':0.32
996744,Cricetomys_gambianus:32.99359470)'321':12.27558280,(Rhizomys_pruinosus:33.01629400,Nannospal
ax_galili:33.01629400)'311':12.25288350)'293':9.53321458,((Jaculus_jaculus:21.42367875,Allactaga_bullata:2
1.42367875)'343':12.20888500,Zapus_hudsonius:33.63256375)'346':21.16982833)'342':15.09598458,((Dipodo
mys_stephensi:8.57769667,Dipodomys_ordii:8.57769667)'340':54.67670833,Castor_canadensis:63.25440500)'
350':6.64397167)'353':0.64655123,(((Xerus_inauris:34.70000000,(Ictidomys_tridecemlineatus:8.62525500,Mar
mota_flaviventris:8.62525500)'349':26.07474500)'339':13.58726214,Aplodontia_rufa:48.28726214)'358':10.708
11619,(Muscardinus_avellanarius:34.61368625,(Glis_glis:28.36757500,Graphiurus_murinus:28.36757500)'361'
:6.24611125)'357':24.38169208)'365':11.54954956)'369':2.33178026)'368':9.26409073)'364':7.68238853,(((((N
ycticebus_coucang:37.78748762,Otolemur_garnettii:37.78748762)'356':21.53688150,(Daubentonia_madagasca
riensis:54.69862455,((Propithecus_coquereli:37.78263867,(Cheirogaleus_medius:35.30000000,(Microcebus_m
urinus:18.44713286,Mirza_coquereli:18.44713286)'338':16.85286714)'292':2.48263867)'377':0.58134765,((Pro
lemur_simus:13.74576625,Lemur_catta:13.74576625)'375':5.76807625,(Eulemur_fulvus:7.66000000,(Eulemur
_macaco:1.16190000,Eulemur_flavifrons:1.16190000)'383':6.49810000)'391':11.85384250)'389':18.85014382)'
399':16.33463823)'398':4.62574457)'397':14.51252523,((((Pithecia_pithecia:17.99321625,Plecturocebus_donac
ophilus:17.99321625)'396':3.88631819,((((Saguinus_imperator:13.90999714,Callithrix_jacchus:13.90999714)'3
94':4.47385933,Aotus_nancymaae:18.38385647)'388':1.29720005,(Saimiri_boliviensis:16.07046167,(Cebus_al
bifrons:1.88944000,Cebus_capucinus:1.88944000)'408':14.18102167)'415':3.61059486)'414':0.98253988,(Alou
atta_palliata:14.89033571,Ateles_geoffroyi:14.89033571)'420':5.77326069)'419':1.21593804)'413':21.27176190
,((((((Mandrillus_leucophaeus:4.59037182,Cercocebus_atys:4.59037182)'412':7.80962818,(Papio_anubis:5.080
28789,Theropithecus_gelada:5.08028789)'425':7.31971211)'411':0.00000000,((Macaca_mulatta:3.68603545,Ma
caca_fascicularis:3.68603545)'407':1.59212755,Macaca_nemestrina:5.27816300)'405':7.12183700)'387':1.3495
7083,((Erythrocebus_patas:5.88566500,Chlorocebus_sabaeus:5.88566500)'431':6.47181833,Cercopithecus_neg
lectus:12.35748333)'429':1.39208750)'436':5.67273395,(((((Rhinopithecus_roxellana:2.67843333,Rhinopithecu
s_bieti:2.67843333)'434':4.48288394,Pygathrix_nemaeus:7.16131727)'428':0.57531416,Nasalis_larvatus:7.736
63143)'386':2.23842786,Semnopithecus_entellus:9.97505929)'382':4.04763833,(Piliocolobus_tephrosceles:12.8

0000000,Colobus_angolensis:12.80000000)'380':1.22269762)'374':5.39960716)'441':10.01924203,(Nomascus_leucogenys:20.18921354,((Gorilla_gorilla:9.06309552,((Pan_troglodytes:2.82005943,Pan_paniscus:2.82005943)'439':3.83084557,Homo_sapiens:6.65090500)'373':2.41219052)'291':6.69907002,Pongo_abelii:15.76216554)'457':4.42704801)'463':9.25233327)'466':13.70974953)'462':23.90975605,Carlito_syrichta:67.06105240)'461':6.77584195)'471':1.86153010,Galeopterus_variegatus:75.69842444)'470':6.40378264,Tupaia_belangeri:82.10220708)'460':7.72098034)'456':6.63920175)'477':8.99739162,(((Tolypeutes_matacus:51.20000000,Chaetophractus_vellerosus:51.20000000)'475':14.81169357,((Tamandua_tetradactyla:13.06000000,Myrmecophaga_tridactyla:13.06000000)'455':42.47476000,(Bradypus_variegatus:34.80363333,(Choloepus_didactylus:6.95000000,Choloepus_hoffmanni:6.95000000)'481':27.85363333)'480':20.73112667)'454':10.47693357)'487':34.49466976,(((Trichechus_manatus:65.48442308,(Procavia_capensis:10.16830000,Heterohyrax_brucei:10.16830000)'485':55.31612308)'453':0.83241222,Loxodonta_africana:66.31683529)'452':16.97210756,((((Microgale_talazaci:43.10000000,Echinops_telfairi:43.10000000)'451':26.89726667,Chrysochloris_asiatica:69.99726667)'495':4.53590833,Elephantulus_edwardii:74.53317500)'493':2.25329167,Orycteropus_afer:76.78646667)'492':6.50247619)'450':17.21742048)'503':4.95341746)'501':53.13780679)'507':18.32991064,Ornithorhynchus_anatinus:176.92749821);

References

1. Lord, E. *et al.* Pre-extinction Demographic Stability and Genomic Signatures of Adaptation in the Woolly Rhinoceros. *Current Biology* (2020) doi:10.1016/j.cub.2020.07.046.
2. Camacho, C. *et al.* BLAST : architecture and applications. *BMC Bioinformatics* vol. 10 421 (2009).
3. Website. http://www. repeatmasker.org/RepeatModeler.html.
4. Website. Smit A, Hubley R, Green P: RepeatMasker Open-3.0. In. http://www.repeatmasker.org.
5. Ersmark, E. *et al.* Population Demography and Genetic Diversity in the Pleistocene Cave Lion. *Open Quaternary* vol. 1 4 (2015).
6. Meyer, M. & Kircher, M. Illumina sequencing library preparation for highly multiplexed target capture and sequencing. *Cold Spring Harb. Protoc.* **2010**, db.prot5448 (2010).
7. Kircher, M., Sawyer, S. & Meyer, M. Double indexing overcomes inaccuracies in multiplex sequencing on the Illumina platform. *Nucleic Acids Res.* **40**, e3 (2012).
8. Briggs, A. W. *et al.* Removal of deaminated cytosines and detection of in vivo methylation in ancient DNA. *Nucleic Acids Res.* **38**, e87 (2010).
9. Website. SeqPrep 1.1. https://github.com/jstjohn/ SeqPrep.
10. Palkopoulou, E. *et al.* Complete genomes reveal signatures of demographic and genetic declines in the woolly mammoth. *Curr. Biol.* **25**, 1395–1400 (2015).
11. Kircher, M. Analysis of High-Throughput Ancient DNA Sequencing Data. *Methods in Molecular Biology* 197–228 (2012) doi:10.1007/978-1-61779-516-9_23.
12. Li, H. & Durbin, R. Fast and accurate long-read alignment with Burrows-Wheeler transform. *Bioinformatics* **26**, 589–595 (2010).
13. Li, H. *et al.* The Sequence Alignment/Map format and SAMtools. *Bioinformatics* **25**, 2078–2079 (2009).
14. Bolger, A. M., Lohse, M. & Usadel, B. Trimmomatic: a flexible trimmer for Illumina sequence data. *Bioinformatics* **30**, 2114–2120 (2014).
15. McKenna, A. *et al.* The Genome Analysis Toolkit: a MapReduce framework for analyzing

next-generation DNA sequencing data. *Genome Res.* **20**, 1297–1303 (2010).

16. Li, H. *et al.* The Sequence Alignment/Map format and SAMtools. *Bioinformatics* vol. 25 2078–2079 (2009).
17. Quinlan, A. R. & Hall, I. M. BEDTools: a flexible suite of utilities for comparing genomic features. *Bioinformatics* **26**, 841–842 (2010).
18. Purcell, S. *et al.* PLINK: a tool set for whole-genome association and population-based linkage analyses. *Am. J. Hum. Genet.* **81**, 559–575 (2007).
19. Korneliussen, T. S., Albrechtsen, A. & Nielsen, R. ANGSD: Analysis of Next Generation Sequencing Data. *BMC Bioinformatics* **15**, 356 (2014).
20. Vieira, F. G., Lassalle, F., Korneliussen, T. S. & Fumagalli, M. Improving the estimation of genetic distances from Next-Generation Sequencing data. *Biological Journal of the Linnean Society* vol. 117 139–149 (2016).
21. Lefort, V., Desper, R. & Gascuel, O. FastME 2.0: A Comprehensive, Accurate, and Fast Distance-Based Phylogeny Inference Program. *Mol. Biol. Evol.* **32**, 2798–2800 (2015).
22. Patterson, N., Price, A. L. & Reich, D. Population structure and eigenanalysis. *PLoS Genet.* **2**, e190 (2006).
23. Price, A. L. *et al.* Principal components analysis corrects for stratification in genome-wide association studies. *Nature Genetics* vol. 38 904–909 (2006).
24. Alexander, D. H., Novembre, J. & Lange, K. Fast model-based estimation of ancestry in unrelated individuals. *Genome Res.* **19**, 1655–1664 (2009).
25. Li, H. & Durbin, R. Inference of human population history from individual whole-genome sequences. *Nature* **475**, 493–496 (2011).
26. Mays, H. L., Jr *et al.* Genomic Analysis of Demographic History and Ecological Niche Modeling in the Endangered Sumatran Rhinoceros Dicerorhinus sumatrensis. *Curr. Biol.* **28**, 70–76.e4 (2018).
27. Roth, T. L. *et al.* Sexual maturation in the Sumatran rhinoceros (Dicerorhinus sumatrensis). *Zoo Biol.* **32**, 549–555 (2013).
28. Prado-Martinez, J. *et al.* Great ape genetic diversity and population history. *Nature* **499**, 471–475

(2013).

29. Haubold, B., Pfaffelhuber, P. & Lynch, M. mlRho - a program for estimating the population mutation and recombination rates from shotgun-sequenced diploid genomes. *Mol. Ecol.* **19 Suppl 1**, 277–284 (2010).

30. Lynch, M. Estimation of nucleotide diversity, disequilibrium coefficients, and mutation rates from high-coverage genome-sequencing projects. *Mol. Biol. Evol.* **25**, 2409–2419 (2008).

31. R Development Core Team. *The R Reference Manual: Base Package*. (Network Theory., 2003).

32. Renaud, G., Hanghøj, K., Korneliussen, T. S., Willerslev, E. & Orlando, L. Joint Estimates of Heterozygosity and Runs of Homozygosity for Modern and Ancient Samples. *Genetics* **212**, 587–614 (2019).

33. Pemberton, T. J. *et al.* Genomic patterns of homozygosity in worldwide human populations. *Am. J. Hum. Genet.* **91**, 275–292 (2012).

34. Davydov, E. V. *et al.* Identifying a high fraction of the human genome to be under selective constraint using GERP++. *PLoS Comput. Biol.* **6**, e1001025 (2010).

35. Hedges, S. B., Marin, J., Suleski, M., Paymer, M. & Kumar, S. Tree of life reveals clock-like speciation and diversification. *Mol. Biol. Evol.* **32**, 835–845 (2015).

36. Cingolani, P. *et al.* A program for annotating and predicting the effects of single nucleotide polymorphisms, SnpEff: SNPs in the genome of Drosophila melanogaster strain w1118; iso-2; iso-3. *Fly* **6**, 80–92 (2012).

37. Trapnell, C. *et al.* Differential gene and transcript expression analysis of RNA-seq experiments with TopHat and Cufflinks. *Nat. Protoc.* **7**, 562–578 (2012).

38. Roberts, A., Trapnell, C., Donaghey, J., Rinn, J. L. & Pachter, L. Improving RNA-Seq expression estimates by correcting for fragment bias. *Genome Biol.* **12**, R22 (2011).

39. R Development Core Team. *The R Reference Manual: Base Package*. (Network Theory, 1999).

40. Reynolds, J., Weir, B. S. & Cockerham, C. C. Estimation of the coancestry coefficient: basis for a short-term genetic distance. *Genetics* **105**, 767–779 (1983).

41. Yi, X. *et al.* Sequencing of 50 human exomes reveals adaptation to high altitude. *Science* **329**, 75–78 (2010).

42. Fumagalli, M. *et al.* Greenlandic Inuit show genetic signatures of diet and climate adaptation. *Science* **349**, 1343–1347 (2015).

43. Mi, H., Muruganujan, A., Casagrande, J. T. & Thomas, P. D. Large-scale gene function analysis with the PANTHER classification system. *Nat. Protoc.* **8**, 1551–1566 (2013).

44. Liu, G. E., Matukumalli, L. K., Sonstegard, T. S., Shade, L. L. & Van Tassell, C. P. Genomic divergences among cattle, dog and human estimated from large-scale alignments of genomic sequences. *BMC Genomics* **7**, 140 (2006).
