# Supplementary material for: Genomic insights into the conservation status of the world’s last remaining Sumatran rhinoceros populations
Source: Nat Commun. 2021 Apr 26;12:2393. doi: 10.1038/s41467-021-22386-8 (PMC8071806; doi:10.1038/s41467-021-22386-8)
Supplement: Supplementary file 3 — Reporting Summary [file 41467_2021_22386_MOESM3_ESM.pdf]

nature research

Corresponding author(s): Johanna von Seth
Nicolas Dussex
Love Dalén

Last updated by author(s): 16/02/21

Reporting Summary

Nature Research wishes to improve the reproducibility of the work that we publish. This form provides structure for consistency and transparency in reporting. For further information on Nature Research policies, see our Editorial Policies and the Editorial Policy Checklist.

Statistics

For all statistical analyses, confirm that the following items are present in the figure legend, table legend, main text, or Methods section.

n/a	Confirmed	
☐	☒	The exact sample size (*n*) for each experimental group/condition, given as a discrete number and unit of measurement
☐	☒	A statement on whether measurements were taken from distinct samples or whether the same sample was measured repeatedly
☐	☒	The statistical test(s) used AND whether they are one- or two-sided<br>*Only common tests should be described solely by name; describe more complex techniques in the Methods section.*
☒	☐	A description of all covariates tested
☐	☒	A description of any assumptions or corrections, such as tests of normality and adjustment for multiple comparisons
☐	☒	A full description of the statistical parameters including central tendency (e.g. means) or other basic estimates (e.g. regression coefficient) AND variation (e.g. standard deviation) or associated estimates of uncertainty (e.g. confidence intervals)
☐	☒	For null hypothesis testing, the test statistic (e.g. *F*, *t*, *r*) with confidence intervals, effect sizes, degrees of freedom and *P* value noted<br>*Give P values as exact values whenever suitable.*
☒	☐	For Bayesian analysis, information on the choice of priors and Markov chain Monte Carlo settings
☒	☐	For hierarchical and complex designs, identification of the appropriate level for tests and full reporting of outcomes
☒	☐	Estimates of effect sizes (e.g. Cohen's *d*, Pearson's *r*), indicating how they were calculated

Our web collection on statistics for biologists contains articles on many of the points above.

Software and code

Policy information about availability of computer code

Data collection	All analyzed data was generated through laboratory procedures, thus no software was used for data collection.
Data analysis	BLAST+ v.2.5.0<br>RepeatModeler v.1.0.8<br>RepeatMasker v.4.0.7<br>https://github.com/tvdvalk/find_CpG<br>bcl2Fastq v2.17.1<br>SeqPrep v.1.1.<br>BWA v.0.7.13<br>SAMtools v.1.3<br>https://github.com/pontussk/samremovedup<br>Trimmomatic v.0.32<br>Picard v.1.141<br>GATK IndelRealigner v.3.4.0<br>BEDTools v. v.2.27.1<br>PLINK v.1.9<br>ANGSD v.0.92<br>ngsDist https://github.com/fgvieira/ngsDist<br>FASTME v.2.0<br>EIGENSOFT v.5.0:<br>https://github.com/mathii/gdc.git<br>ADMIXTURE v.1.3.0

PSMC v.0.6.5
mlRho v.2.7
ROHan: https://github.com/grenaud/ROHan
R v.3.3.3
GERP++
TimeTree: http://www.timetree.org/
htsbox v.1.0: https://github.com/lh3/htsbox
SnpEff v.2.3
Cufflinks v.2.2.1
Panther v.16.0

For manuscripts utilizing custom algorithms or software that are central to the research but not yet described in published literature, software must be made available to editors and reviewers. We strongly encourage code deposition in a community repository (e.g. GitHub). See the Nature Research guidelines for submitting code & software for further information.

Data

Policy information about availability of data

All manuscripts must include a data availability statement. This statement should provide the following information, where applicable:

- Accession codes, unique identifiers, or web links for publicly available datasets
- A list of figures that have associated raw data
- A description of any restrictions on data availability

- D. sumatrensis harrissoni assembly: https://www.ncbi.nlm.nih.gov/bioproject/PRJNA638009/, BioProjectID: PRJNA638009, Assembly: GCA_014189135.1
- C. simum simum assembly: https://www.ncbi.nlm.nih.gov/assembly/GCF_000283155.1/, BioProjectID: PRJNA74583, assembly: GCF_000283155.1
- Historical and modern resequencing data (fastq files): ENA (https://www.ebi.ac.uk/ena/browser/view/PRJEB35511, project accession number PRJEB35511)
- Published mammalian reference genomes downloaded for the estimation of mutational load based on evolutionary constrained regions are available at NCBI (https://www.ncbi.nlm.nih.gov/assembly/organism/40674/all/)
- Figures that have associated raw data:

Figure 1. Sampling, current range, and phylogeny of Sumatran rhinoceros
Figure 2. Population history and timing of population divergence for Sumatran rhinoceros
Figure 3. Inbreeding and mutational load estimates in modern Sumatran rhinoceros populations
Figure 4. Temporal changes in inbreeding and mutational load estimates

Field-specific reporting

Please select the one below that is the best fit for your research. If you are not sure, read the appropriate sections before making your selection.

☒ Life sciences ☐ Behavioural & social sciences ☐ Ecological, evolutionary & environmental sciences

For a reference copy of the document with all sections, see nature.com/documents/nr-reporting-summary-flat.pdf

Life sciences study design

All studies must disclose on these points even when the disclosure is negative.

Sample size	21 newly sequenced historical and modern genomes. Samples size was determined and limited by both modern and historical specimen availability.
Data exclusions	Historical data was filtered for quality using standard practices in the field of ancient DNA. No data was completely excluded, but samples with an average genome coverage <8X was only included for population structure analyses (PCA and ADMIXTURE).
Replication	This study did not involve any controlled experiments, therefore no replication was performed. Sampling was limited by both modern and historical specimen availability. Sampling was conducted from all extant wild populations and from all individuals approved by the ethical permits, so replication was not possible.<br><br>All methods used are described in detail, so that the analyses can be reproduced by other researchers by accessing the raw data deposited in ENA (see entries under 'Data').
Randomization	This study did not involve any controlled experiments, therefore no randomization was performed.<br><br>While different types of tissues can have different DNA yield after extraction, we adjusted our sequencing efforts accordingly with the goal of having an average genome coverage >8X. Samples that didn't yield enough DNA to reach corresponding average coverage were only included for population structure analyses (PCA and admixture).<br><br>Samples were grouped by population and/or time period for analysis of population structure (PCA, ADMIXTURE), and for the comparison of heterozygosity (mlRho), inbreeding (FROH), mutational load (GERP analysis and SnpEff analysis), and local adaptation (SnpEff and PBS analysis).
Blinding	This study did not involve any controlled experiments, so no blinding was performed.

Reporting for specific materials, systems and methods

We require information from authors about some types of materials, experimental systems and methods used in many studies. Here, indicate whether each material, system or method listed is relevant to your study. If you are not sure if a list item applies to your research, read the appropriate section before selecting a response.

Materials & experimental systems

n/a	Involved in the study	
x		Antibodies
	x	Eukaryotic cell lines
x		Palaeontology and archaeology
	x	Animals and other organisms
x		Human research participants
x		Clinical data
x		Dual use research of concern

Methods

n/a	Involved in the study	
x		ChIP-seq
x		Flow cytometry
x		MRI-based neuroimaging

Eukaryotic cell lines

Policy information about cell lines

Cell line source(s)	Fibroblast cell lines: 6196, 6197, 6198, 14999, 9243. Fibroblast cell lines used for DNA extraction were obtained from commercial sources. They were all generated at San Diego Zoo Global (SDZG) from biopsy pieces collected from captive animals.
Authentication	All fibroblast cell lines used for DNA extractions were karyotyped as a quality control step to verify their diploid number, as shown in Steiner et al. (2018).<br><br>Steiner, C. C., Houck, M. L., & Ryder, O. A. (2018). Genetic variation of complete mitochondrial genome sequences of the Sumatran rhinoceros (Dicerorhinus sumatrensis). Conservation genetics, 19(2), 397-408.
Mycoplasma contamination	Cell lines were not tested for mycoplasma contamination.
Commonly misidentified lines (See ICLAC register)	No commonly misidentified cell lines were used in this study.

Animals and other organisms

Policy information about studies involving animals; ARRIVE guidelines recommended for reporting animal research

Laboratory animals	No laboratory animals were included in this study.
Wild animals	No animals were killed or captured for this study.<br><br>Four modern D. sumatrensis harrissoni specimens:<br>Gelugob: female, ~35 years old at the time of sampling (post-mortem sampling)<br>Puntung: female, age at sampling not known but deceased at an age of ~20-25 years<br>Kertam: male, age at sampling not known but deceased at an age of ~30 years<br>Iman: female, age at sampling not known but deceased at an age of ~25 years<br><br>Sampling of Gelugob, Puntung, Kertam, and Iman was conducted when the animals had already been transported to rhino sanctuaries or nature reserves, during routine health monitoring or, in the case of Gelugob that had deceased due to circumstances not related to this study, post-mortem. Sampling was conducted by Borneo Rhino Alliance (BORA) and the Wildlife Rescue Unit.<br><br>--<br><br>Eleven modern D. sumatrensis sumatrensis and one D. sumatrensis harrissoni specimens:<br>KB6196: female, birth date not known<br>KB6197: female, birth date not known<br>KB6198: female, estimated birth date 1984<br>KB7902: male, birth date 1990<br>KB8031: female, estimated birth date 1984<br>KB9200: female, estimated birth date 1988<br>KB9218: male, birth date 1992<br>KB20219: female, birth date 2004<br>KB8126: female, estimated birth date 1984<br>KB9342: female, estimated birth date 1988

KB14999: female, estimated birth date 1983
OR2142: sex not known, birth date not known

Specific details on sampling of the eleven modern D. sumatrensis sumatrensis and one D. sumatrensis harrissoni is lacking, but were conducted in captivity in Melaka Zoo, Malaysia (KB6196, KB6197, KB6198), San Diego Zoo, USA (KB7902, KB8031, KB9200, KB9218), Cincinnati Zoo, USA (KB20219, KB8126, KB9342), NY Bronx Zoo, USA (KB14999), and by Sabah Wildlife Department, in Borneo, Malaysia (OR2142).

Field-collected samples

Blood (Puntung, Kertam, Iman, and Gelugob) and tissue samples (Gelugob) were placed in BD Vacutainer Plastic K3EDTA tubes immediately after sampling, and subsequently stored in room temperature until DNA extraction.

A detailed description of sampling of the eleven modern D. sumatrensis sumatrensis and one D. sumatrensis harrissoni is lacking.

Ethics oversight

Utilization of samples was compliant with applicable regulatory procedures for CITES and the US Endangered Species Act.

Export of blood and tissue samples for DNA extraction and genome sequencing from Sumatran rhinoceros individuals from Sabah to the Swedish Museum of Natural History, Sweden, was approved by The Sabah Biodiversity Council and the director of Sabah Wildlife Department in 2014 (Licence Ref JKM/MBS.1000-2/2 (373, CITES import/export permit numbers 51138-14 / 0736).

Exports of DNA extracts for genome sequencing from the San Diego Zoo Global Frozen Zoo®, USA, to the Swedish Museum of Natural History, Sweden, was between two CITES-registered institutions (COSE transfer, CITES exemption reference number 30-3314/99), as well as under Mutual Transfer Agreement (request number BR2016005).

Note that full information on the approval of the study protocol must also be provided in the manuscript.
